# Inducing Fe 3*d* Electron Delocalization and Spin-State Transition of FeN_4_ Species Boosts Oxygen Reduction Reaction for Wearable Zinc–Air Battery

**DOI:** 10.1007/s40820-023-01014-8

**Published:** 2023-02-10

**Authors:** Shengmei Chen, Xiongyi Liang, Sixia Hu, Xinliang Li, Guobin Zhang, Shuyun Wang, Longtao Ma, Chi-Man Lawrence Wu, Chunyi Zhi, Juan Antonio Zapien

**Affiliations:** 1 Department of Materials Science and Engineering, City University of Hong Kong, Hong Kong, 999077 People’s Republic of China; 2 Sustech Core Research Facilities, Southern University of Science and Technology, 1088 Xueyuan Blvd, Shenzhen, Guangdong 518055 People’s Republic of China; 3 Frontiers Science Center for Flexible Electronics, Institute of Flexible Electronics, Northwestern Polytechnical University, Xi’an, 710072 People’s Republic of China; 4 Tsinghua Shenzhen International Graduate School, Tsinghua University, Shenzhen, Guangdong, 518055, People’s Republic of China

**Keywords:** Fe 3*d* electron delocalization, Spin-state transition, Oxygen reduction reaction, Wearable zinc–air batteries

## Abstract

Transition metal–nitrogen–carbon materials (M–N–Cs), particularly Fe–N–Cs, have been found to be electroactive for accelerating oxygen reduction reaction (ORR) kinetics. Although substantial efforts have been devoted to design Fe–N–Cs with increased active species content, surface area, and electronic conductivity, their performance is still far from satisfactory. Hitherto, there is limited research about regulation on the electronic spin states of Fe centers for Fe–N–Cs electrocatalysts to improve their catalytic performance. Here, we introduce Ti_3_C_2_ MXene with sulfur terminals to regulate the electronic configuration of FeN_4_ species and dramatically enhance catalytic activity toward ORR. The MXene with sulfur terminals induce the spin-state transition of FeN_4_ species and Fe 3*d* electron delocalization with d band center upshift, enabling the Fe(II) ions to bind oxygen in the end-on adsorption mode favorable to initiate the reduction of oxygen and boosting oxygen-containing groups adsorption on FeN_4_ species and ORR kinetics. The resulting FeN_4_–Ti_3_C_2_S_*x*_ exhibits comparable catalytic performance to those of commercial Pt-C. The developed wearable ZABs using FeN_4_–Ti_3_C_2_S_*x*_ also exhibit fast kinetics and excellent stability. This study confirms that regulation of the electronic structure of active species via coupling with their support can be a major contributor to enhance their catalytic activity.

**Supplementary Information:**

The online version contains supplementary material available at 10.1007/s40820-023-01014-8.

## Highlights

- The strong interaction between Ti_3_C_2_S_x_ and FeN_4_ species induces the central metal Fe(II) in FeN_4_ species with intermediate spin state transferred to high spin state, in which the latter is favorable to initiate the reduction of oxygen.
- This strong interaction induces a remarkable Fe 3*d* electron delocalization with *d* band center upshift, boosting oxygen-containing groups adsorption on FeN_4_ species and oxygen reduction reaction kinetics.
- The resulting FeN_4_–Ti_3_C_2_S_*x*_ with FeN_4_ moieties in high spin state exhibits high half-wave potential of 0.89 V vs. RHE and high limiting current density of 6.5 mA cm^−2^, enabling wearable zinc–air battery showing a good discharge performance with a maximum power density of 133.6 mW cm^−2^.

**Supplementary Information:**

The online version contains supplementary material available at 10.1007/s40820-023-01014-8.

## Graphical abstract

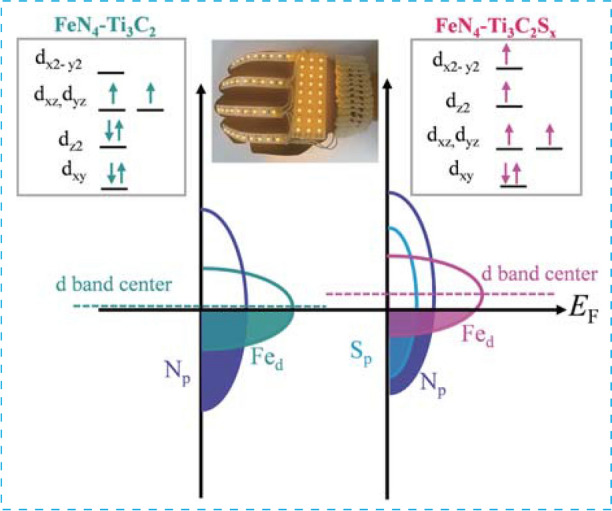

## Introduction

The growing energy demands stimulates intense research on renewable and economical energy technologies, including fuel cells and metal–air batteries [1–3]. A key technical limitation associated with them is the sluggish kinetics of oxygen reduction reaction (ORR) involved in the air cathode [4]. To date, substantial efforts have been developed to explore suitable catalysts to improve the ORR kinetics [5–9]. The benchmark catalysts are precious metal-based materials; however, their high cost and poor stability have largely prevented their large-scale applications [10–12]. Fortunately, a wide range of dispersed metals anchored on nitrogen-doped carbons (M–N–Cs) have been found to be electroactive for ORR [13–20]. Fe–N–Cs hold particular attention owing to the multifarious electronic structure of FeN_4_ moieties [21–23].

To date, massive efforts have been developed to incorporate FeN_4_ species, improve their density, and design geometric structures for FeN_4_ species exposed [24, 25]. Though previous works have proved that regulation on electronic structure of FeN_4_ species such as manipulation of the metal center, atomic vacancy, and edge defect can improve their activity, the electron configuration of Fe(II) in such FeN_4_ species has been considered for different spin states: (i) low spin state with electron configuration of *d*_*xy*_^2^*d*_*yz*_^2^*d*_*xz*_^2^; (ii) intermediate spin state with electron configuration of *d*_*xy*_^2^*d*_*yz*_^1^*d*_*xz*_^1^*d*_*zz*_^2^; and (iii) high spin state with electron configuration of *d*_*xy*_^2^*d*_*yz*_^1^*d*_*xz*_^1^*d*_*z*2_^1^*d*_*x*2-*y*2_^1^ [26, 27]. Some researchers suppose both the low spin and high spin states are favorable to catalyze ORR reaction because an empty 3*d*_z2_ orbital or occupied by a single electron enables Fe(II) ions to bind oxygen in the end-on adsorption mode which is more readily to absorb and desorb related reaction intermediates and thus facilitate ORR catalysis. Some researchers consider that the intermediate spin state with fully filled 3*d*_*z*2_ orbital prevents the end-on adsorption of oxygen on Fe(II) ions, which hinders the reduction of oxygen on this site [26–29]. However, the exact correlation between the electronic spin configuration of the active site and the ORR activity has remained inadequately understood, which impedes the rational design of high-performance ORR catalysts. Thus, directly regulating the electronic spin states of Fe centers for FeN_4_ species’ to study the ORR catalytic activity is necessary. To achieve this, it is promising to have a catalyst support with good electronic conductivity and mechanical stability for FeN_4_ species dispersion and capable of inducing an interaction with the active species [30].

MXenes, as a new kind of 2D materials, are fabricated by selective extraction of A layer from M_*n*+1_AX_*n*_ phase (where M stands for Ti, V, Nb, Mo, etc.; A represents Al, Si, Ga, etc.; X is C or N; *n* = 1–3) [31–33]. In light of electronegativity, surface hydrophilicity, good mechanical stability, and electronic conductivity, MXenes emerge as a fascinating catalyst support [31–33]. In particular, the easy surface tunability of MXenes enables the easy tailoring ability of near-MXene surface environment and their supported catalyst [31]. Thus, it is attractive to apply MXenes as FeN_4_ species support. However, to modify the electronic structure of FeN_4_ active species, further tailoring the MXenes structure is necessary. Incorporating sulfur terminal has been proved to adjust the surface polarities and electronic properties of carbon-based materials successfully [34–36]. Therefore, we suppose that incorporating S in the MXenes terminal can also tailor the electronic properties of MXenes, enabling the modification of the electronic structure on the supported FeN_4_ active species owing to the strong interaction between the active species and the MXenes support.

Herein, we realize a significant improvement for the intrinsic ORR activity of Fe–N–Cs electrocatalyst via introducing the sulfur-terminated Ti_3_C_2_ MXene as the support to disperse the iron–nitrogen species. The resulting catalyst is denoted as FeN_4_–Ti_3_C_2_S_x_. We perform X-ray absorption fine spectroscopy (XAFS) and X-ray photoelectron spectroscopy (XPS) measurements on this catalyst to disclose that the iron is coordinated with nitrogen in the form of FeN_4_, while the sulfur in Ti_3_C_2_S_*x*_ terminal is bond with N to manipulate the electronic structure of central Fe sites. Furthermore, such FeN_4_ coupling withTi_3_C_2_S_x_ leads to a remarkable Fe 3*d* electron delocalization with d band center upshift and the central metal Fe(II) in FeN_4_ species from original intermediate spin state (*d*_*xy*_^2^*d*_*yz*_^1^*d*_*xz*_^1^*d*_*z*2_^2^) transferring to high spin state (*d*_*xy*_^2^*d*_*yz*_^1^*d*_*xz*_^1^*d*_*z*2_^1^*d*_*x*2- y2_^1^) confirmed via ultraviolet photoelectron spectroscopy (UPS), electron spin resonance (ESR) spectroscopy, temperature-dependent magnetic susceptibility (M − T) measurements, and density functional theory (DFT) calculations. A *d*_*z*2_ orbital occupied by a single electron enables their Fe(II) ions to bind oxygen in the end-on adsorption mode which is more readily to absorb and desorb related reaction intermediates and thus facilitates ORR catalysis. Besides, the remarkable Fe 3*d* electron delocalization with d band center upshift can optimize the orbital hybridization of Fe 3*d* with *p* orbital of oxygen-containing groups, boosting oxygen-containing groups adsorption on FeN_4_ species and ORR kinetics. Our catalyst exhibits remarkable catalytic performance enhancement with positively shifted of 80 mV for half-wave potential compared with the one without sulfur terminals, named FeN_4_–Ti_3_C_2_. Furthermore, its catalytic activity is comparable to that of commercial 20% Pt-C for half-wave potential and limiting current density (half-wave potential: 0.89 V vs. RHE for FeN_4_ –Ti_3_C_2_S_*x*_, 0.88 V vs. RHE for Pt-C; limiting current density: 6.5 mA cm^−2^ for FeN_4_ -Ti_3_C_2_S_*x*_, 5.5 mA cm^−2^ for Pt–C) and its long-term stability is superior to that of commercial Pt-C. Besides, integrating this FeN_4_–Ti_3_C_2_S_*x*_ catalyst into a wearable ZAB shows a good discharge performance with a maximum power density of 133.6 mW cm^−2^ and a high cycling stability with 110 h at 2 mA cm^−2^, demonstrating the feasibility of FeN_4_–Ti_3_C_2_S_*x*_ in ZAB applications.

## Experimental Section

### Materials

LiF powder (99.99%), Ti_3_AlC_2_ MAX powder (90%), FeCl_3_ (97%), and KSCN (99%), and 2,2'-bipyridine were supplied by Sigma-Aldrich. Concentrated sulfuric acid (H_2_SO_4_, ≥ 98 wt%) and hydrochloric acid (HCl, 35 wt%) were supplied from Shanghai Aladdin Bio-Chem Technology Co., LTD (China).

### Preparation of Ti_3_C_2_ MXene and FeN_4_–Ti_3_C_2_S_***x***_

#### Synthesis of Ti_3_C_2_ MXene

The Ti_3_C_2_ MXene was obtained using a wet chemical method reported by Gogotsi [37]. Typically, 1 g of LiF powder was dissolved in 10 mL of 9 M HCL via stirring for 30 min at room temperature. Then, 1 g of Ti_3_AlC_2_ MAX powder was added slowly into the above solution under ice bath condition, stirred the sealing mixed solution continuously for 24 h at 35 °C, washed this mixture solution with DI water 10 times, sonicated the resulting sediment in DI for 20 min, and then centrifuged at 3500 rpm for 20 min, and the obtained black colloidal supernatant was the Ti_3_C_2_ MXene solution. The Ti_3_C_2_ MXene was obtained via freeze drying the black colloidal supernatant for 48 h.

#### Synthesis of FeN_4_–Ti_3_C_2_S_x_

For FeN_4_–Ti_3_C_2_S_x_ synthesis, 1 mmol of FeCl_3_ and 3 mmol of KSCN were dissolved in 25 mL of DI water via vigorous stirring for 30 min. Then, 50 mg of the as-prepared Ti_3_C_2_ MXene dissolved in 25 mL enthanol was added into the above solution and stirred for another 30 min; then sonicated the mixed solution for 1 h; after that, added 2 mmol 2, 2'-bipyridine in the above mixture solution and continuously stirred for 20 h at 25 °C until getting a dark red slurry; and next, collected the slurry and dried at 80 °C in vacuum. Then, the dried precursor was ground in quartz mortar and annealed at 950 °C at a heating rate of 5 °C min^−1^ under Ar atmosphere for 2 h. Finally, the annealed product was soaked in 0.5 M H_2_SO_4_ at 80 °C for 8 h to remove inactive iron species. The leached sample was washed to neutral with water and enthanol for three times and dried in vacuum at 60 °C for 24 h. The FeN_4_–Ti_3_C_2_ reference sample was obtained via the similar processes without adding 3 mmol of KSCN.

### Characterizations

#### Materials Characterization

The methodology and structure of the prepared materials were evaluated via using scanning electron microscopy (SEM) (Philips XL30 FEG) and transmission electron microscopy (TEM, FEI Tecnai G2 F30 with 300 kV of accelerating voltage). The crystal structure was analyzed by using X-ray diffraction (Bruker, D2 Phaser) with Cu Kα (λ = 1.5418 Å) radiation and Raman spectroscopy (Renishaw in Via™ confocal Raman microscope with a excitation laser of 514 nm wavelength). The specific surface area was derived from the N_2_ adsorption–desorption isotherms obtained with a Micrometric ASAP 2020 instrument. Besides, the Barrett–Joyner–Halenda (BJH) method was applied to obtain the pore size distribution. The chemical composition was evaluated by using X-ray photoelectron spectroscopy (XPS) (VG ESCALAB 220i-XL). The chemical coordinated information was obtained by X-ray absorption fine spectroscopy (XAFS) spectra conducted at the beamline 1W1B of Beijing Synchrotron Radiation Facility (BSRF) at Institute of High Energy Physics, Chinese Academy of Sciences. The storage rings of BSRF were conducted at 2.5 GeV with an average current of 250 mA. The data collection was conducted in transmission mode using ionization chamber when using Si (111) double-crystal monochromator. The data were processed and analyzed similar to previous procedures via using ATHENA and ARTEMIS for X-ray absorption near-edge structure (XANES) and the extended X-ray absorption fine structure (EXAFS) spectra, respectively [38]. Ultraviolet photoemission spectroscopy (UPS) measurements were carried out on an ESCA LAB 250 Xi spectrometer with He I resonance lines (21.2 eV). Temperature-dependent magnetic susceptibility (M*-*T) measurements were conducted in the temperature range from 10 to 300 K with a physical property measurement system model 6000 (Quantum Design, USA). Electron spin resonance (ESR) spectra were obtained by an ER200-SRC-10/12 (Bruker, Germany) spectrometer at 300 K.

#### Electrochemical Measurements

All electrochemical measurements were conducted on a CHI 760E electrochemical workstation integrating a rotating ring disk electrode (RRDE) in a three electrodes system, in which a glassy carbon electrode (GCE) with diameter (3 mm) loaded with catalyst as working electrode, while Pt sheet and Ag/AgCl (3 M KCl) were used as counter electrode and reference electrode, respectively. The recorded potential was converted to reversible hydrogen electrode (RHE) potential according to the following equation: *E*_RHE_ = *E*_Ag/Ag/Cl_ + 0.059 × pH + 0.210. The loading mass was 280 ug cm^−2^ for all of the catalysts measured.

##### ORR Measurements

All electrochemical measurements were operated in the N_2_ or O_2_ saturated 0.1 M KOH electrolyte. The cyclic voltammetry (CV) measurements were recorded at a scan rate of 100 mv s^−1^ in N_2_ or O_2_ saturated electrolyte. The LSV measurements were performed at a scan rate of 5 mV s^−1^ in O_2_ saturated electrolyte. Each catalyst repeated at least 3 times of each measurement to exclude possible incidental errors. The numbers (*n*) of electron transferred per O_2_ molecule were calculated according to the following Koutecky–Levich (K*-*L) Eq. 1 [39]:

1 $$\frac{1}{j} = \frac{1}{{j_{k} }} + \frac{1}{{B\omega^{0.5} }}$$

where *j* is the measured electrode current density,* j*_*k*_ is the kinetic current density, and *ω* is theelectrode rotating rate. *B* represents the Levich slope given by the following Eq. 2:

2 $$B = 0.2n{\text{F}}({\text{D}}_{{{\text{O}}_{2} }} )^{{{2 \mathord{\left/ {\vphantom {2 3}} \right. \kern-0pt} 3}}} \nu^{{{{ - 1} \mathord{\left/ {\vphantom {{ - 1} 6}} \right. \kern-0pt} 6}}} {\text{C}}_{{{\text{O}}_{2} }}$$

where *n* is the number of electrons transferred per oxygen molecule, F is the Faraday constant (F = 96,485 C mol^−1^), D_O2_ is the diffusion coefficient of O_2_ in 0.1 M KOH, where D_O2_ is 1.9 × 10^–5^ cm^2^ s^−1^, v is the kinetic viscosity (0.01 cm^2^ s^−1^), and C_O2_ is the bulk concentration of O_2_, which is 1.2 × 10^–6^ mol cm^−3^. Constant 0.2 is used when the rotating speed is expressed in rpm.

The RRDE tests were performed using a Pt ring surrounded by 4 mm diameter GCE. The value was determined by the following Eq. 3:

3 $$n = \frac{{4i_{d} }}{{i_{d} + \frac{{i_{r} }}{{\text{N}}}}}$$

The HO_2_^−^ yield is decided by Eq. 4:

4 $${\text{HO}}_{2}^{ - } {\text{\% }} = \frac{{200i_{r} }}{{{\text{Ni}}_{d} + i_{r} }}$$

where *i*_*d*_ represents the disk current and *i*_*r*_ represents the ring current. N represents the current collection efficiency of the Pt ring which is determined as 0.44.

The long-term stability was analyzed by chronoamperometric tests at a fixed potential of 0.7 V vs. RHE and a rotation speed of 1600 rpm in O_2_ saturated electrolyte.

##### Electrochemical Double Layer Capacitances (C_dl_)

The C_dl_ was measured via a simple CV method. The C_dl_ of various materials can be determined from the CV measurement, which is expected to be linearly proportional to the electrochemical active surface areas. A potential range of −0.1–0.1 V vs. Ag/AgCl was selected for measuring capacitance because no obvious Faradaic currents were observed in this region. The absolute value capacitive currents of ΔJ@0 V/2 were plotted as a function of the CV scan rate of 20, 40, 60, 80, and 100 mV s^−1^. The slopes of the fitting data line were the geometric C_dl_.

##### Electrochemical Impedance Spectroscopy (EIS)

The EIS measurements were carried out by applying an AC voltage with 5 mV amplitude in a frequency range from 100 to 100 mHz.

##### Aqueous Zinc–Air Battery (ZAB) Assembly

The air electrode was fabricated via spraying catalyst slurry on a clean carbon cloth with an active area of 1 cm^2^ and then dried at room temperature for 24 h. The catalyst slurry was obtained via dispersing 8 mg of FeN_4_–Ti_3_C_2_S_*x*_ or Pt–C catalysts into 1 ml of mixed solution containing 2-propanol, DI water, and Nafion solution (5 wt%) with a ratio of 10:40:3. The resulting loading mass was 1.0 mg cm^−2^. The air electrode served as cathode, 6.0 M KOH with 0.2 M Zn(Ac)_2_ addictive as electrolyte, and polished Zn plate electrode as anode to assemble an aqueous rechargeable ZAB.

##### Stretchable Solid-State Fiber-Shaped ZAB Assembly

The air electrode was assembled via spraying catalyst slurry on a carbon nanotube (CNT) paper with a loading mass of 1.0 mg cm^−2^ and then dried at room temperature for 24 h. The dual-network PANa-cellulose hydrogel was synthesized using our previous developed method and soaking with 6.0 M KOH with 0.2 M Zn(Ac)_2_ addictive as stretchable solid-state electrolyte. The anode was a zinc spring. The stretchable solid-state fiber-shaped ZAB was assembled via the following process: (a) coated the relaxed Zn spring anode with dual-network PANa-cellulose hydrogel electrolyte; (b) stretched the spring-hydrogel system; and (c) coated the catalyst loading CNT paper on the stretched spring-hydrogel system, then released. Due to strong adhesion of hydrogel, the anode and cathode can be firmly adhered to hydrogel electrolyte. The galvanostatic tests were performed via using a Land 2001 A battery test system at room temperature. The charge–discharge polarization and A.C. impedance with 5 mV amplitude in a frequency range from 100 to 100 mHz were determined by using an electrochemical workstation (CHI 760e, Chenhua)**.**

### Computational Details

All the first principle calculations were conducted using spin-polarized DFT as implemented in Quantum Espresso [40, 41]. Generalized gradient approximation (GGA) with Perdew–Burke–Ernzerhof (PBE) functional was chosen to represent the exchange–correlation interaction [42]. Grimme’s DFT-D3 method was used to determine van der Waals (vdW) interactions [43]. A plane-wave cutoff of 55 Ry and a density cutoff of 550 Ry were applied based on standard solid-state pseudopotentials with projector augmented-wave (PAW) method [44, 45]. The DFT + U calculations were adopted to describe strong on-site Coulomb interaction of localized electrons. The value of Hubbard correction U for 3*d* orbitals of Ti and Fe was set to 3.0 and 5.0 eV, respectively. A 4 × 4 supercell of MXene monolayer with a sufficiently vacuum slab of 20 Å was built. For sampling the Brillouin zone, Monkhorst–Pack *k*-point was set as 2 × 2 × 1, and a larger 6 × 6 × 1 *k*-point was applied to study the electronic properties. All atoms were fully relaxed until the forces on each atom were less than 0.02 eV Å^−1^.

The calculation of Gibbs free energy change (ΔG) for each elemental step was based on the computational hydrogen electrode (CHE) model [46], which could be expressed by ΔG = Δ*E* + Δ*E*_ZPE_—*T*Δ*S* + *e*U + ΔG_pH_, where ∆*E* represents the electronic energy difference between the free standing and adsorption states of reaction intermediates; ∆*E*_ZPE_ and ∆*S* represent the changes in zero point energies and entropy, respectively, which are obtained from the vibrational frequency calculations. *T* represents the temperature and here is set as 298.15 K; *e* and U represent the number of electrons transferred and the electrode applied potential, respectively; ∆G_pH_ represents the free energy correction of pH, which can be derived from: ∆G_pH_ = K_*B*_*T* × pH × ln10. In this work, H_2_ and H_2_O were used as the reference states; hence, a series of equivalent reactions for the ORR mechanism are applied to determine ΔG. The complete ORR catalytic process in alkaline condition includes the following five elementary steps [22]:

(i) O_2_(*g*) + * → O_2_*
(ii) O_2_* + H_2_O + *e*¯ → OOH* + OH¯
(iii) OOH* + *e*¯ → O* + OH¯
(iv) O* + H_2_O + *e*¯ → OH* + OH¯
(v) OH* + *e*¯ → OH¯

where * indicates the adsorption site and steps ii − v represent the four-electron transfer processes.

## Results and Discussion

### Catalysts Fabrication and Structural Characterizations

**Fig. 1:**
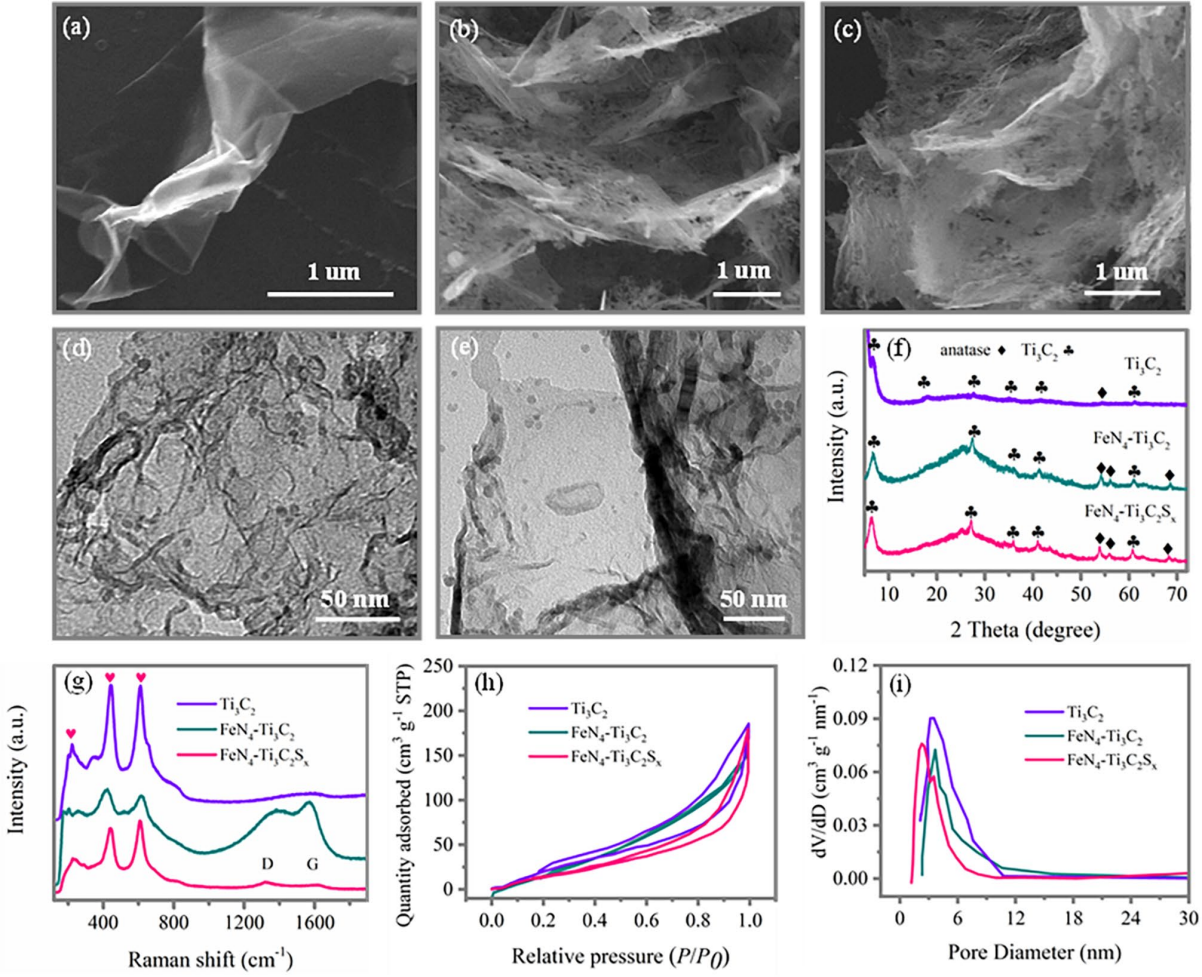
SEM images for **a** pristine Ti_3_C_2_, **b** FeN_4_–Ti_3_C_2_, and **c** FeN_4_–Ti_3_C_2_S_*x*_. TEM images for **d** FeN_4_–Ti_3_C_2_, and **e** FeN_4_–Ti_3_C_2_S_*x*_. **f** XRD patterns, and **g** Raman spectra. **h** N_2_ adsorption–desorption isotherms, and corresponding **i** pore size distribution of the products

The FeN_4_–Ti_3_C_2_S_x_ sample was obtained by coating Ti_3_C_2_ MXene with Fe salt, 2,2-bipyridine and potassium thiocyanate (KSCN), followed by pyrolysis at 900 °C in N_2_ atmosphere and HF acid leaching process. FeN_4_–Ti_3_C_2_ sample was obtained via similar method except that KSCN was not used. The SEM image of Ti_3_C_2_ MXene, as shown in Fig. 1a, indicates that it is nanosheet structure with wrinkle, while after coating with Fe, N, and S salts before carbonization, it becomes thicker as indicated in Fig. S1. After carbonization, when the Fe, N, S dopant are introduced, the products become thicker and rough and few nanoparticles attached to the surface are clearly seen for FeN_4_–Ti_3_C_2_ and FeN_4_–Ti_3_C_2_S_*x*_ in Fig. 1b, c, respectively. The morphology of these two samples, without and with *S* dopant, is similar as also confirmed by the TEM images indicated in Fig. 1d for FeN_4_–Ti_3_C_2_, and in Fig. 1e for FeN_4_–Ti_3_C_2_S_*x*_. The XRD patterns in Fig. 1f show the peaks at 2θ = 6.7°, 17.8°, and 27.6°, which are characteristic of (002), (006), and (008) crystal planes of layered pristine Ti_3_C_2_ MXene, respectively [47]. Besides, the peaks of other two samples with Fe, N, and *S* dopants are similar to those of pristine Ti_3_C_2_, indicating that introducing dopant does not change the structure of pristine Ti_3_C_2_; however, more anatase peaks appear indicating the doping process resulting in mild oxidation of Ti_3_C_2_ flakes, which is also consistent with the SEM results that some nanoparticles appear in the rough surface of Ti_3_C_2_ after introducing dopant. Besides, the broad peak at ~ 25° suggests the formation of amorphous carbon during the doping process owing to the mid oxidation of Ti_3_C_2_ flakes. The Raman spectra in Fig. 1g indicate that the peaks of the samples with Fe, N, and S dopants are similar to those of pristine Ti_3_C_2_; however, the peak intensity of the carbon skeleton (D/G bands) becomes more obvious in the spectra for samples with dopants compared with pristine Ti_3_C_2_. This is because more defects generated during dopant and carbonization process. Normally, the defects could affect the catalytic performance by influencing the electronic conductivity and active sites of samples [35, 48]. Figure 1h shows the N_2_ adsorption and desorption isotherms of all three samples displaying a type IV isotherm with hysteresis, suggesting the existence of porosities in the structure. The calculated BET surface areas of these samples follow the trend of Ti_3_C_2_ > FeN_4_–Ti_3_C_2_ > FeN_4_–Ti_3_C_2_S_*x*_, and mesoporous structures are dominant in all three samples as shown in Fig. 1i. The reduced BET surface areas and pore diameter as the increasing of the dopants’ species may be caused by the dopants filling effect.

**Fig. 2:**
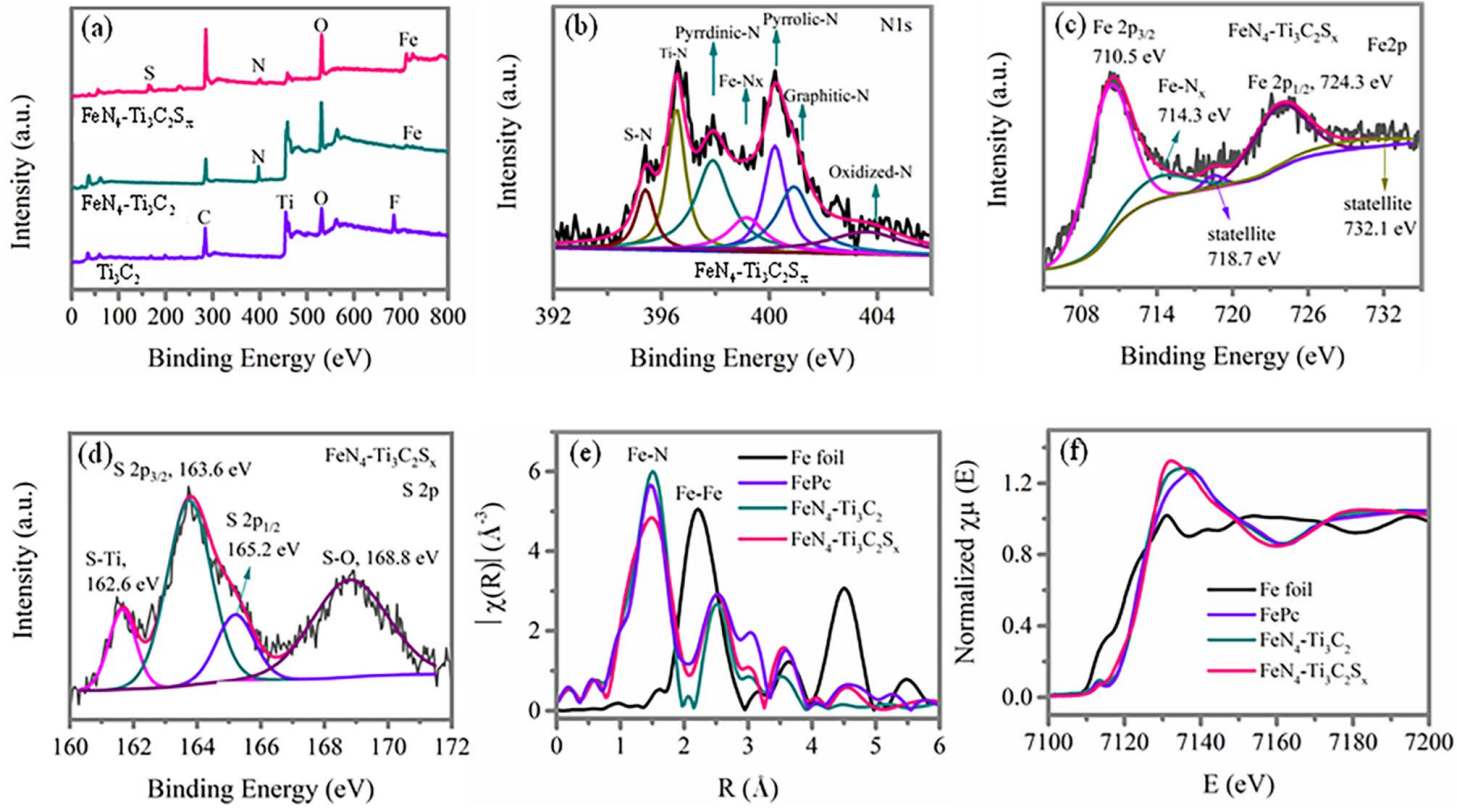
**a** XPS survey spectra of pristine Ti_3_C_2_, FeN_4_–Ti_3_C_2_, and FeN_4_–Ti_3_C_2_S_*x*_. High-resolution XPS spectra of **b** N 1*s*, **c** Fe 2*p*, and **d** S 2*p* for sample FeN_4_–Ti_3_C_2_S_*x*_. **e** Fourier transforms of Fe K-edge EXAFS spectroscopy oscillations of samples FeN_4_–Ti_3_C_2_, and FeN_4_–Ti_3_C_2_S_*x*_, with Fe foil and FePc as references. **f** Fe K-edge XANES spectra of samples FeN_4_–Ti_3_C_2_, and FeN_4_–Ti_3_C_2_S_*x*_, with Fe foil and FePc as references

The surface compositions of the prepared samples have been analyzed by XPS, as presented in Fig. 2. The survey XPS spectra in Fig. 2a show that the F 1*s*, O 1*s*, Ti 2*p*, and C 1*s* peaks exhibit in pristine Ti_3_C_2_, while F 1*s* is absent in samples FeN_4_–Ti_3_C_2_ and FeN_4_–Ti_3_C_2_S_*x*_, suggesting that during dopants introduced process, the surface F atoms in pristine Ti_3_C_2_ disappear. Instead, the N 1*s* and Fe 2*p* peaks exhibit in sample FeN_4_–Ti_3_C_2_ and FeN_4_–Ti_3_C_2_S_*x*_, suggesting that the Fe and N atoms are introduced in the pristine Ti_3_C_2_ MXene. Besides, we can see that the S 2*p* peak only exhibits in sample FeN_4_ -Ti_3_C_2_S_x_, indicating that only sample FeN_4_–Ti_3_C_2_S_*x*_ has S atom as expected. The corresponding elementary composition obtained from XPS survey spectra of sample FeN_4_–Ti_3_C_2_ and FeN_4_–Ti_3_C_2_S_*x*_ is provided in Table S1. The high-resolution XPS spectrum of N 1*s* for sample FeN_4_–Ti_3_C_2_S_*x*_ shown in Fig. 2b shows a peak at around 396.5 eV that can be assigned to Ti–N species. The Ti–N peaks in high-resolution XPS spectra of N 1*s* (Fig. S2a) for sample FeN_4_–Ti_3_C_2_ are similar to those in sample FeN_4_–Ti_3_C_2_S_*x*_. The peak at around 395.4 eV presented in sample FeN_4_–Ti_3_C_2_S_*x*_ may relate to S coordinated with N atom species, denoted as S–N species here, since this peak is absent in sample FeN_4_–Ti_3_C_2_ without sulfur dopant. Besides, the observation of Fe–N_*x*_ peak centered at 399.1 eV in N 1*s* of these two samples suggests that the N atoms are coordinated with Fe atoms instead of *S* atoms coordinating with Fe atoms [22]. The high-resolution XPS spectra of Fe 2*p* for samples FeN_4_–Ti_3_C_2_S_*x*_ (Fig. 2c) and FeN_4_–Ti_3_C_2_ (Fig. S2b) can be fitted to four peaks, with two spin–orbit doublets at around 710.5 and 724.3 eV, which corresponds to the Fe 2*p*_3/2_ and Fe 2*p*_1/2_, respectively. Besides, the peak at around 714.3 eV is assigned to the Fe–N_*x*_ configuration, which further confirms that the Fe atoms are coordinated with N atoms in these two samples [1, 49–51]. The high-resolution XPS spectrum of S 2*p* for sample FeN_4_–Ti_3_C_2_S_*x*_ as shown in Fig. 2d can be fitted to four peaks at binding energies of 162.6, 163.6, 165.2, and 168.8 eV, while the peak at 162.6 eV is attributed to Ti–S bond, indicating that the sulfur atoms substitute the terminal groups in the pristine Ti_3_C_2_ MXene and form S–Ti–C bond in sample FeN_4_–Ti_3_C_2_S_*x*_ [52, 53]. Since no peak related to S coordinated with Fe can be observed, the exotic S atoms tend to bond to surrounding N atoms instead directly to the Fe atoms. The peaks at 163.8 and 165.2 eV correspond to S 2*p*_3/2_ and S 2*p*_1/2_ of the C–S–C covalent bond of thiophene-S. The peak at 168.8 eV is assigned to oxidized sulfur [54]. The high-resolution XPS spectra of Ti 2*p* for sample FeN_4_–Ti_3_C_2_ (Fig. S2c) and sample FeN_4_–Ti_3_C_2_S_*x*_ (Fig. S2c) can be fitted to four doublets, including two peaks assigned to Ti–C, two assigned to Ti^2+^, two assigned to Ti^3+^, and two assigned to Ti–O, in agreement with previous reports [55].

To further identify the coordinated status of Fe, N, and S atoms in these two samples, X-ray absorption near-edge structure (XANES) and Fourier transform extended X-ray absorption fine structure (EXAFS) spectrometry measurements are conducted. Figure 2e shows the Fourier transforms EXAFS at the Fe K-edge of samples FeN_4_–Ti_3_C_2_, FeN_4_–Ti_3_C_2_S_*x*_, commercial FePc (iron phthalocyanine), and Fe foil samples as references, which reveals that samples FeN_4_–Ti_3_C_2_ and FeN_4_–Ti_3_C_2_S_*x*_ exhibit a primary strong peak at ~ 1.5 Å, corresponding to Fe–N peak, originating from a nitrogen shell surrounding iron atoms in reference to that of FePc [27, 56]. The Fe-centered coordination number and bond distance of these samples derived from EXAFS fitting is shown in Fig. S3 and Table S2 and confirms the coordination number of FeN_*x*_ for samples FeN_4_–Ti_3_C_2_ and FeN_4_–Ti_3_C_2_S_*x*_ is ~ 4 which is also similar to that of sample FePc. This confirms that the presence of FeN_4_ moieties in samples FeN_4_–Ti_3_C_2_ and FeN_4_–Ti_3_C_2_S_*x*_ and FeN_4_ species is usually considered as the main active species for catalysis ORR [57–59]. The phase-corrected bond length of Fe–N shell is increased from 1.92 to 1.97 Å from sample FePc to FeN_4_–Ti_3_C_2_ and then further increased to 1.98 Å for FeN_4_–Ti_3_C_2_S_*x*_ but shorter than that of Fe-S (2.15 Å), suggesting only Fe–N in these two samples. This also indicates that the square-planar structure of Fe^2+^ − N_4_ moieties becomes distorted in sample FeN_4_–Ti_3_C_2_ compared with that in commercial FePc, which is further distorted in sample FeN_4_–Ti_3_C_2_S_*x*_. Besides, the Fe–Fe peaks at ~ 2.2 Å are only presented in sample Fe foil, indicating no iron-based crystalline structures in both samples FeN_4_–Ti_3_C_2_ and FeN_4_–Ti_3_C_2_S_*x*_ [28, 60]. Figure 2f shows the XANES spectra at the Fe K-edge of samples FeN_4_–Ti_3_C_2_, FeN_4_–Ti_3_C_2_S_*x*_, and the commercial FePc, Fe foil samples as references; an enlarged version of this figure, with added y-axis offset, is included as Fig. S4 for clarity. These figures show that sample FePc presents a weak pre-edge peak at ~ 7113.3 eV, assigning to a fingerprint of square-planar Fe^2+^ − N_4_ moieties [22]. This feature becomes less obvious but is still perceptible in sample FeN_4_–Ti_3_C_2_; however, it is not perceptible in sample FeN_4_–Ti_3_C_2_S_*x*_ suggesting that the square-planar structure of Fe^2+^ − N_4_ moieties becomes distorted in sample FeN_4_–Ti_3_C_2_, which is destructed in sample FeN_4_–Ti_3_C_2_S_*x*_ [26]. The distortion of the square-planar structure of Fe^2+^ − N_4_ moieties is caused by the distortion of the D_4h_ symmetry as a result of the central Fe away from the N_4_-plane due to the interaction of the Ti_3_C_2_ support while incorporation of S terminals further enhances the interaction resulting in destruction of the D_4h_ symmetry and square-planar structure [27].

### ORR Performance

**Fig. 3:**
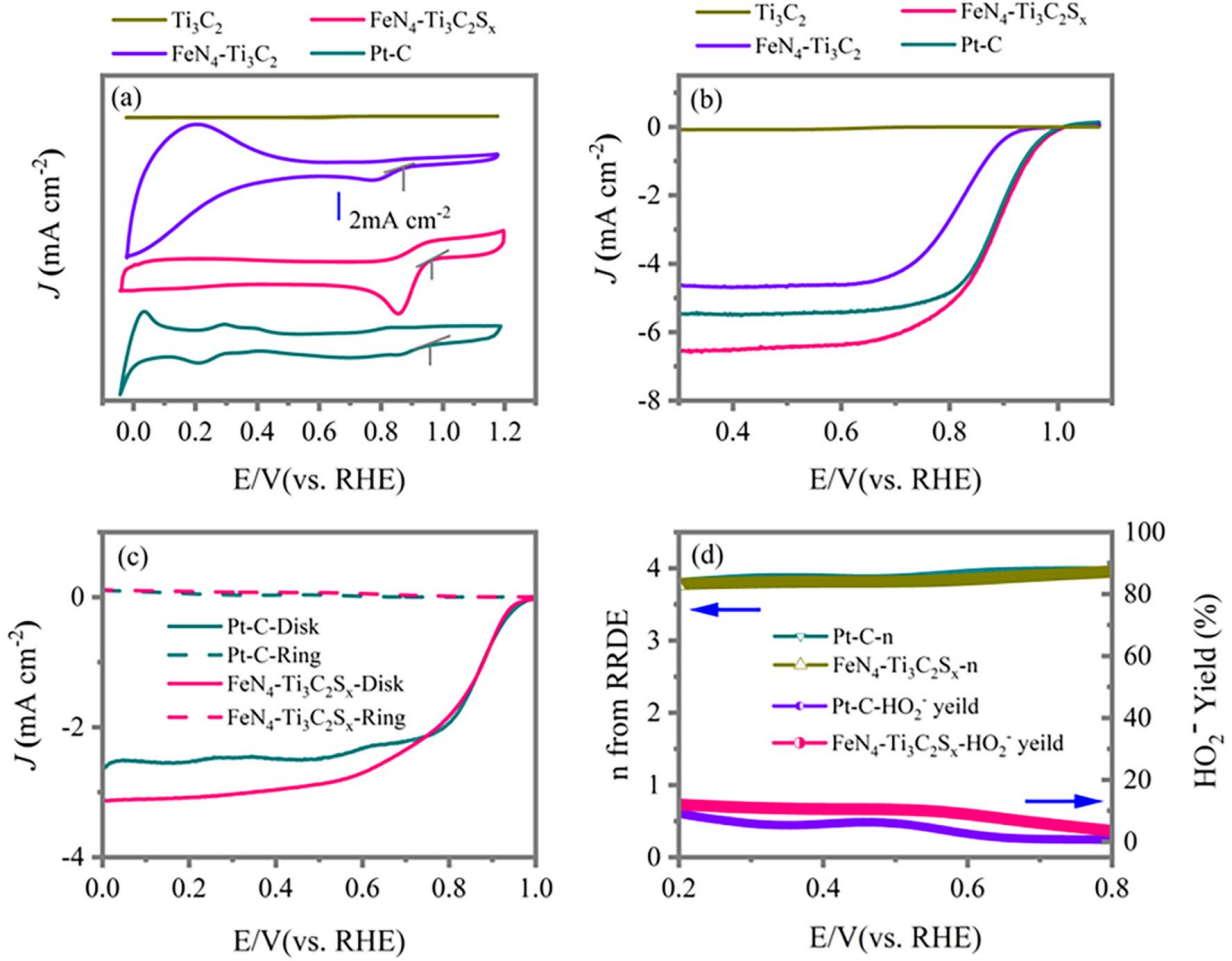
**a** CV curves of pristine Ti_3_C_2_, FeN_4_–Ti_3_C_2_, FeN_4_–Ti_3_C_2_S_*x*_, and commercial Pt–C recorded at 100 mV s^−1^ in O_2_ saturated 0.1 M KOH solution. **b** LSV curves of pristine Ti_3_C_2_, FeN_4_–Ti_3_C_2_, FeN_4_–Ti_3_C_2_S_*x*_, and commercial Pt–C at 1600 rpm rotation speeds. **c** RRDE measurements of samples FeN_4_–Ti_3_C_2_S_*x*_, and commercial Pt–C at 1600 rpm. **d** Electron transfer numbers (*n*) and HO_2_^−^ yield derived from RRDE tests

To investigate the ORR activity of the obtained materials, we conducted cyclic voltammetry (CV) and liner sweep voltammetry (LSV) measurements. The CV curves, recorded in O_2_ saturated 0.1 M KOH, are presented in Fig. 3a and show that the oxygen reduction peak potential for pristine Ti_3_C_2_ is 0.60 V vs. RHE (its enlarged version in Fig. S5c), indicating that pristine Ti_3_C_2_ presents poor ORR activity. After the introduction of Fe and N dopants, the peak potential has been improved to 0.77 V vs. RHE for sample FeN_4_–Ti_3_C_2_. Interestingly, with additional S terminals, the peak potential has been further improved to 0.862 V vs. RHE for sample FeN_4_–Ti_3_C_2_S_x_, which is comparable to that of commercial Pt-C (0.860 V). However, the pristine Ti_3_C_2_ with only S terminal (Ti_3_C_2_S_*x*_) does not show obvious enhancement when compared with pristine Ti_3_C_2_ (Fig. S5a). This suggests that the sample FeN_4_–Ti_3_C_2_S_*x*_ processed the highest ORR activity ORR activity. This phenomenon is also proved in the LSV curves recorded at a rotation speed of 1600 rpm, as indicated in Fig. 3b. The half-wave potential for pristine Ti_3_C_2_ is 0.64 V vs. RHE (its enlarged version in Fig. S5d), while for FeN_4_–Ti_3_C_2_ it is improved to 0.81 V vs. RHE. A more positive half-wave potential of 0.89 V vs. RHE for sample FeN_4_–Ti_3_C_2_S_*x*_ is observed, and the improvement is up to 80 mV of half-wave potential after introducing S terminals. Besides, the corresponding Tafel plots as shown in Fig. S5b also indicate that sample FeN_4_–Ti_3_C_2_S_*x*_ possesses the smallest Tafel slope, suggesting the highest ORR kinetics. However, the half-wave potential and Tafel slope of Ti_3_C_2_S_*x*_ are almost similar to those of pristine Ti_3_C_2_. We also conduct electrochemical double layer capacitance (C_dl_) and electrochemical impedance spectroscopy (EIS) measurements for the above samples to compare the electrochemical active surface areas and the charge transfer resistance. The Cdl for such samples are shown in Fig. S6, suggesting the electrochemical active surface area trend is: Ti_3_C_2_ ˂ Ti_3_C_2_S_*x*_˂ FeN_4_–Ti_3_C_2_ ˂ FeN_4_–Ti_3_C_2_S_*x*_. The semicircular diameters of samples Ti_3_C_2_, Ti_3_C_2_S_*x*_, FeN_4_–Ti_3_C_2_, and FeN_4_–Ti_3_C_2_S_*x*_ follow the trend of Ti_3_C_2_ > Ti_3_C_2_S_*x*_ > FeN_4_–Ti_3_C_2_ > FeN_4_–Ti_3_C_2_S_*x*_ as shown in Fig. S7, suggesting the interface charge transfer resistance of such samples follow the trend of Ti_3_C_2_ > Ti_3_C_2_S_x_ > FeN_4_–Ti_3_C_2_ > FeN_4_–Ti_3_C_2_S_*x*_. Both the C_dl_ and EIS results are consistent with the that of the catalytic activity. The high performance of sample FeN_4_ -Ti_3_C_2_S_x_ is comparable to that of commercial Pt–C (0.88 V) and outperforms many other related electrocatalysts reported in recent literatures (Table S3). The rotating disk electrode (RDE) tests recorded at various rotation speeds are further performed to obtain more ORR kinetics information as indicated in Fig. S8. It is obvious that each sample FeN_4_–Ti_3_C_2_ (Fig. S8b), FeN_4_–Ti_3_C_2_S_*x*_ (Fig. S8e), and Pt–C (Fig. S8h) shows well-defined diffusion-limited platforms at rotation speed from 400 to 3600 rpm. The corresponding Koutecky–Levich plots (K*-*L plots) at various potentials exhibit good linearity. The calculated average electron transfer number (*n*) is 3.64 for sample FeN_4_–Ti_3_C_2_, 3.97 for sample FeN_4_–Ti_3_C_2_S_*x*_, and 3.99 for commercial Pt–C, as shown in Fig. S8c, f, and i, respectively. The electron transfer numbers of sample FeN_4_–Ti_3_C_2_S_*x*_ and Pt-C are highly close to 4, indicating a four-electron reduction pathway. The parallel rotating ring disk electrode (RRDE) tests shown in Fig. 3c, d show that the electrons transfer numbers of sample FeN_4_–Ti_3_C_2_S_*x*_ locate between 3.81 and 3.97 with a HO_2_^−^ yield of 2.1–11.9%, which are close to those of commercial Pt–C (*n*: 3.81–3.99; HO_2_^−^ yield: 0.7–9.1%). In addition, the long-term stability indicated in Fig. S9 indicates that sample FeN_4_–Ti_3_C_2_S_*x*_ retains 84% of its initial current, which surpasses that of commercial Pt–C with 52% of its initial current retention in a continuous 24 h test, manifesting a better cycling stability for sample FeN_4_–Ti_3_C_2_S_*x*_.

### Wearable Solid-state ZAB Performance

**Fig. 4:**
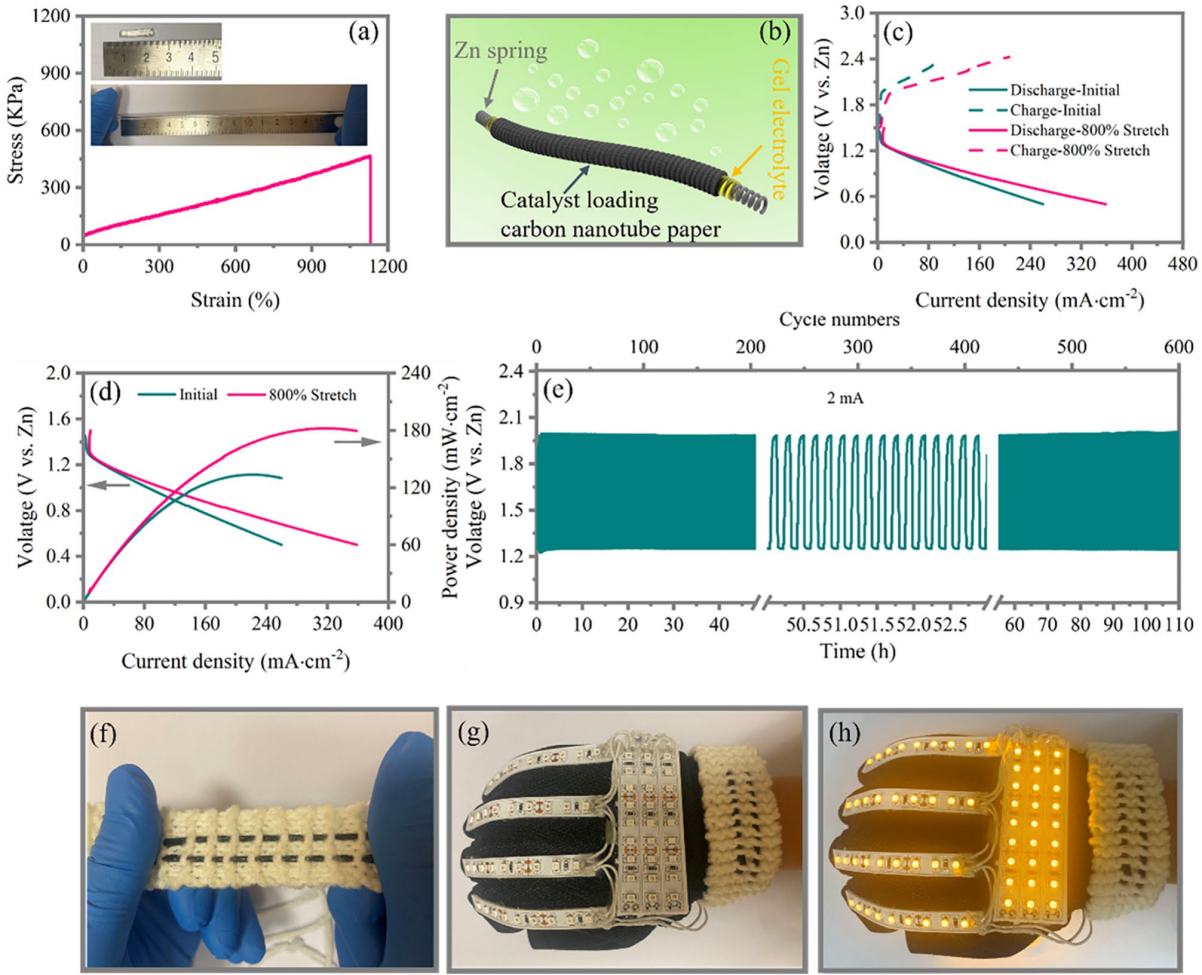
**a** Tensile stress versus strain curve of the prepared PANa-cellulose hydrogel with 6 M KOH + 0.2 M Zn(CH_3_COO)_2_ intake; the insets are optical photos of the initial and stretched states of this hydrogel electrolyte. **b** Schematic illustration of stretchable fiber-shaped ZAB. **c** Galvanodynamic charge–discharge profiles of fiber-shaped ZAB at initial and 800% stretched states. **d** Galvanodynamic discharge and corresponding power density curves of fiber-shaped ZAB at initial and 800% stretched states. **e** Cycling stability test of fiber-shaped ZAB at 2 mA cm^−2^; **f** Photographs of two fiber-shaped ZABs (length: 10 cm, diameter: 2 mm) woven into a wristband; **g** Photographs of this wristband connected to a glove. **h** Photographs of this wristband connected to a glove to power a set of LEDs

Since sample FeN_4_–Ti_3_C_2_S_*x*_ presents high ORR activity, we further investigate the aqueous ZAB performance of this sample and commercial Pt–C–IrO_2_ for comparison, as shown in Fig. S10. Notably, the energy efficiency, maximum power density, and cycling stability of FeN_4_–Ti_3_C_2_S_*x*_-based ZAB are better than those based on commercial Pt–C–IrO_2_ electrodes emphasizing the promising potential of FeN_4_–Ti_3_C_2_S_*x*_ catalyst for rechargeable ZAB applications. In addition, we also construct a stretchable and wearable fiber-shaped ZAB using our developed FeN_4_–Ti_3_C_2_S_*x*_ catalyst and a recently developed alkaline tolerant dual-network PANa and cellulose hydrogel (PANa-cellulose) as stretchable solid-state electrolyte [13]. The stretchability of this PANa-cellulose hydrogel soaked with 6 M KOH + 0.2 M Zn(CH_3_COO)_2;_ Fig. 4a shows that it can be stretched over 1000% strain without any breakage and visible cracking demonstrating excellent stretchability performance. The structure of the fiber-shaped ZAB is depicted in Fig. 4b, using hydrogel electrolyte to coat the Zn spring electrode firstly and then stretch them and finally coat them by using the FeN_4_–Ti_3_C_2_S_*x*_ loading carbon nanotube paper as air electrode. The charge–discharge profiles and corresponding power density of the fiber-shaped ZAB at initial and 800% stretched states are shown in Fig. 4c, d, which indicates that the ZAB in stretched state exhibits increased energy efficiency and power density when compared with those in initial state. The increases in the energy efficiency and power density for the stretched state can be attributed to the increased contact areas between the hydrogel electrolyte and the active materials. The maximum power density for ZAB in initial state is 133.6 mW cm^−2^ and at 800 stretched state is 182.3 mW cm^−2^, suggesting that the battery is stretchable and the electrochemical performance is good under stretched state. Besides, the battery shows excellent cycling stability with 110 h stable cycles at 2 mA cm^−2^, as shown in Fig. 4e. To demonstrate its wearability, two fiber-shaped ZABs with a length of 10 cm and a diameter of 2 mm have been woven into a wristband and connected to a wearing glove, as shown in Fig. 4f, g, respectively. This wristband can power a set of LEDs on the wearing glove demonstrating the feasibility of such an efficient stretchable and wearable fiber-shaped ZABs based on the prepared FeN_4_–Ti_3_C_2_S_*x*_ catalyst.

### Origin of the ORR Electrocatalytic Activity

**Fig. 5:**
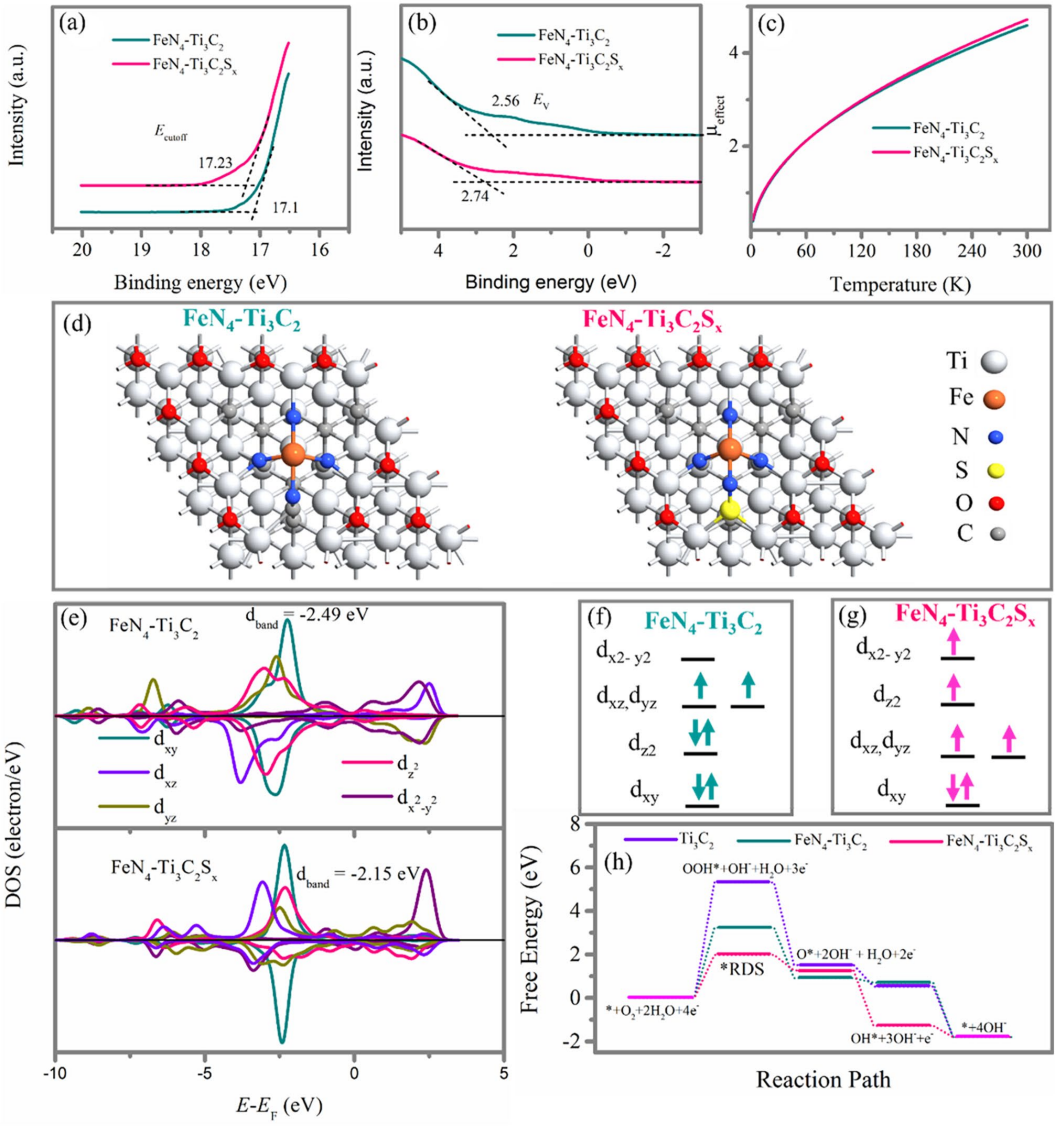
UPS spectra of samples FeN_4_–Ti_3_C_2_, and FeN_4_–Ti_3_C_2_S_*x*_: **a** in the normalized secondary electron cutoff energy *E*_cutoff_ regions, and **b** the valence band maximum *E*_V_ regions. **c** The effective magnetic moment *µ*_effect_ for samples FeN_4_–Ti_3_C_2_, and FeN_4_–Ti_3_C_2_S_*x*_, obtained from M–T measurements. **d** Top view of computational optimized atomic structures of FeN_4_–Ti_3_C_2_ (left) and FeN_4_–Ti_3_C_2_S_*x*_ (right). **e** PDOS of the Fe center in samples FeN_4_–Ti_3_C_2_ (above), and FeN_4_–Ti_3_C_2_S_*x*_ (below). To better present the location of energy states around the Fermi level, Fermi level is always defined to be 0 eV. Schematic representation of the electronic spin state of partial Fe(II) on **f** FeN_4_–Ti_3_C_2_, and **g** FeN_4_–Ti_3_C_2_S_*x*_. **h** Free energy diagram for the optimized atomic structures of Ti_3_C_2_, FeN_4_–Ti_3_C_2_, and FeN_4_–Ti_3_C_2_S_*x*_ during ORR under alkaline conditions

The remarkable ORR catalytic activity of our prepared FeN_4_–Ti_3_C_2_S_x_ should come from the possible interactions between the FeN_4_ species and the sulfur-terminated Ti_3_C_2_ support. To verify this interaction, multiple spectroscopies are conducted to explore the electronic structure changes of FeN_4_ species supported on pristine Ti_3_C_2_ MXene between on sulfur terminated Ti_3_C_2_ MXene. UPS is first conducted to study the band structure of samples FeN_4_-Ti_3_C_2_ and FeN_4_-Ti_3_C_2_S_x_. As indicated in Fig. 5a, the cutoff energy (*E*_cutoff_) of FeN_4_–Ti_3_C_2_ is 17.1 while FeN_4_–Ti_3_C_2_S_*x*_ is 17.23. The work function Φ can be calculated according to Φ = *hv*—|*E*_cutoff_—*E*_F_| [30], where *hv* represents the photon energy of the excitation light (21.22 eV) and *E*_F_ here is 0 eV. Therefore, the work functions Φ are estimated as 4.12 eV for FeN_4_–Ti_3_C_2_ and 3.99 eV for FeN_4_–Ti_3_C_2_S_*x*_. Furthermore, the valence band maximum (*E*_V_) shifts to lower energy after the introduction of S into the Ti_3_C_2_ support as shown in Fig. 5b where *E*_V_ is estimated to be 2.56 and 2.74 eV for FeN_4_–Ti_3_C_2_ and FeN_4_–Ti_3_C_2_S_*x*_, respectively. The decrease of Φ and *E*_V_ shift to lower energy for FeN_4_–Ti_3_C_2_S_*x*_ compared to FeN_4_–Ti_3_C_2_ demonstrates that the electrons within FeN_4_ moieties become more spatially stable after incorporation of S terminals in Ti_3_C_2_ support and that the 3*d* band center of Fe (II) changes. This means that the electron density of FeN_4_ moieties distributes more thinly at Fe (II) center, while more densely at the nitrogen ligands, resulting in Fe 3*d* electron delocalization caused by the sulfur terminal with high electronegativity interacting strongly with FeN_4_ moieties and stabilizing the valence band maximum by reducing the electron density of Fe(II). It is believed that a decreased electron density of Fe (II) center with strong delocalization can optimize the orbital overlap of Fe 3*d* with O_2_ 2*p* and thus favors the oxygen adsorption on FeN_4_ species and ORR kinetics [30].

Besides, we have also performed ESR and M*-*T measurements to disclose the electron spin configuration of FeN_4_ moieties in Ti_3_C_2_ support with and without S terminals. The ESR spectra in Fig. S11 indicate an obvious shift and larger g factor for FeN_4_–Ti_3_C_2_S_*x*_ compared with those of sample FeN_4_–Ti_3_C_2_. The shift of ESR spectra is due to stronger interaction between S terminated Ti_3_C_2_ and unpaired electrons in FeN_4_ active species for –Ti_3_C_2_S_*x*_ compared to that of sample FeN_4_–Ti_3_C_2_. The larger g factor for sample FeN_4_–Ti_3_C_2_S_*x*_ is due to more unpaired electrons occurrence caused by the stronger interaction between FeN_4_ species and Ti_3_C_2_S_*x*_ supporter. The M*-*T measurements are presented in Fig. S12. The corresponding effective magnetic moment (*µ*_effect_) shown in Fig. 5c indicates that the *µ*_effect_ of sample FeN_4_–Ti_3_C_2_S_*x*_ is larger than that of sample FeN_4_–Ti_3_C_2_. The larger *µ*_effect_ demonstrates that the larger number of unpaired d electron of Fe(II) in sample FeN_4_-Ti_3_C_2_S_*x*_ compared with that of in sample FeN_4_–Ti_3_C_2_. Both ESR and M*-*T measurements prove the spin state of central Fe(II) in FeN_4_ moieties for sample FeN_4_–Ti_3_C_2_ is higher than that of FeN_4_–Ti_3_C_2_, and thus, we speculate that the central Fe(II) in FeN_4_ moieties for sample FeN_4_–Ti_3_C_2_ may possess an intermediate spin state with electron configuration of *d*_*xy*_^2^*d*_*yz*_^1^*d*_*xz*_^1^*d*_*z*2_^2^, which transfers into high spin state with electron configuration of* d*_*xy*_^2^*d*_*yz*_^1^*d*_*xz*_^1^*d*_*z*2_^1^*d*_*x*2-*y*2_^1^ after introducing S terminals in sample FeN_4_–Ti_3_C_2_S_*x*_.

To gain deeper insight into the electronic structure of FeN_4_ moieties in samples FeN_4_–Ti_3_C_2_ and FeN_4_–Ti_3_C_2_S_*x*_, DFT calculations are performed. The computational details are presented in Experimental Section. The FeN_4_ moieties originally processes the square-planar structure with central Fe(II) in low spin state with electron configuration of *d*_*xy*_^2^*d*_*yz*_^2^*d*_*xz*_^2^. However, this square-planar structure will become distorted with central Fe ion slightly out of N_4_ plane when the FeN_4_ moieties anchored on the Ti_3_C_2_ support, constrained by the structure of Ti_3_C_2_, as shown in Fig. 5d (left). The additional S atom adjacent to FeN_4_ moiety will further pull the central Fe out of N_4_ plane, as shown in Fig. 5d (right). The detail top view and side view of computational optimized atomic structure of pristine Ti_3_C_2_, FeN_4_–Ti_3_C_2_, and FeN_4_–Ti_3_C_2_S_x_ are also indicated in Fig. S13. The calculated PDOS, shown in Fig. 5e (above), indicates that the FeN_4_ moieties in sample FeN_4_–Ti_3_C_2_ exhibit the magnetic moment due to the spin splitting of the partially occupied *d*_*xz*_ and *d*_*yz*_ orbitals of Fe(II). The *d*_*xy*_ and *d*_*z*_^2^ orbitals are fully filled, while the *d*_x_^2^-_y_^2^ is empty. This electron spin configuration in sample FeN_4_–Ti_3_C_2_ corresponds to intermediate spin states of central Fe(II) in FeN_4_ moiety, as indicated in Fig. 5f. Interestingly, introducing S terminals can increase the on-site magnetic moment of Fe center (Fig. S14) and tune the spin state of Fe(II) in FeN_4_ moiety where the spin splitting of the partially occupied *d*_*z*_^2^ and *d*_*x*_^2^-_*y*_^2^ orbitals occur, while the d_*xy*_ orbital is also fully filled. The spin splitting of partially occupied d_*xz*_ and d_*yz*_ orbitals decrease, but still can be observed, as shown in Fig. 5e. This electron spin configuration in sample FeN_4_–Ti_3_C_2_S_*x*_ is close to high spin states of central Fe (II) in FeN_4_ moiety, as indicated in Fig. 5g. Thus, it suggests that the central Fe(II) in FeN_4_ moieties for sample FeN_4_–Ti_3_C_2_ processes an intermediate spin state with electron configuration of *d*_*xy*_^2^*d*_*yz*_^1^*d*_*xz*_^1^*d*_*z*2_^2^, while transfers into high spin state with electron configuration of *d*_*xy*_^2^*d*_*yz*_^1^*d*_*xz*_^1^*d*_*z*2_^1^*d*_*x*2-*y*2_^1^ after introducing S terminals in sample FeN_4_–Ti_3_C_2_S_*x*_, which is consistent with ESR and M*-*T results. The introduction of S terminals affects the configuration way for Fe coordinating with N, resulting in different configuration of *d* orbitals for Fe as shown in Fig. 5f, g, so their energy orders are different [24, 51]. The transfer of Fe(II) electron spin configuration from intermediate spin state in FeN_4_ moieties for sample FeN_4_–Ti_3_C_2_ to high spin state for sample FeN_4_–Ti_3_C_2_S_x_ could enhance the adsorption of molecular oxygen on catalyst surface and thus improve the ORR performance [52–54]. In addition, the calculated PDOS indicates the electron density of Fe atom reduces and the d band center rises from −2.49 to −2.15 eV. The Fe 3*d* electron delocalization and d band center upshift can optimize the orbital hybridization of Fe 3*d* with *p* orbital of oxygen-containing groups, boosting oxygen-containing groups adsorption on FeN_4_ species and ORR kinetics [55–57].

The free energy diagram of ORR processes for pristine Ti_3_C_2_, FeN_4_–Ti_3_C_2_, and FeN_4_–Ti_3_C_2_S_*x*_ in alkaline condition (pH = 13) is shown in Fig. 5h. It is noted that the formation of *OOH is the rate-determining step (RDS) for ORR of these three systems. After the introduction of S terminals, the adsorption of *OOH intermediate on FeN_4_–Ti_3_C_2_S_*x*_ significantly increases, indicating that FeN_4_–Ti_3_C_2_S_*x*_ system exhibits better catalytic activity for ORR, which agrees well with experimental results.

## Conclusions

Conventional strategies to design efficient Fe–N–Cs catalysts are based on incorporating FeN_4_ species, improving their density, and designing geometric structures for exposing FeN_4_ species; however, few works have focused on regulating the electronic structure of FeN_4_ species, especially their electronic spin states for improved activity. We demonstrate that introducing sulfur-terminated Ti_3_C_2_ MXene to support FeN_4_ species via fabricating FeN_4_–Ti_3_C_2_S_x_ sample can regulate the electronic spin state of FeN_4_ species and dramatically enhance catalytic activity toward ORR. Our experimental investigation and theoretical studies uncover that the sulfur-terminated MXene induces the central metal Fe(II) in FeN_4_ species with original intermediate spin state (*d*_*xy*_^2^*d*_*yz*_^1^*d*_*xz*_^1^*d*_*z*2_^2^) transfer to high spin state (*d*_*xy*_^2^*d*_*yz*_^1^*d*_*xz*_^1^*d*_*z*2_^1^*d*_*x*2- y2_^1^) in which the latter the *d*_*z*2_ orbital occupied by a single electron enables their Fe(II) ions to bind oxygen in the end-on adsorption mode favorable to initiate the reduction of oxygen. Furthermore, it induces a remarkable Fe 3*d* electron delocalization with *d* band center upshift, optimizing the orbital hybridization of Fe 3*d* with *p* orbital of oxygen-containing groups, boosting oxygen-containing groups adsorption on FeN_4_ species and ORR kinetics. The resulting FeN_4_–Ti_3_C_2_S_x_ exhibits enhanced 80 mV of half-wave potential compared to that of the FeN_4_–Ti_3_C_2_ and also comparable catalytic performance to those of commercial Pt–C. Besides, integrating this FeN_4_–Ti_3_C_2_S_x_ catalyst into a wearable ZAB shows a good discharge performance and high cycling stability. This study endows a guideline for regulation on electronic structure of active species via coupling with their support, which is significant to enhance catalytic activity.

### Supplementary Information

Below is the link to the electronic supplementary material.

Supplementary file1 (PDF 2101 KB)
