# Supplementary material for: Inducing Fe 3d Electron Delocalization and Spin-State Transition of FeN4 Species Boosts Oxygen Reduction Reaction for Wearable Zinc–Air Battery
Source: Nanomicro Lett. 2023 Feb 10;15:47. doi: 10.1007/s40820-023-01014-8 (PMC9918713; doi:10.1007/s40820-023-01014-8)
Supplement: Supplementary file 1 — Supplementary file1 (PDF 2101 KB) [file 40820_2023_1014_MOESM1_ESM.pdf]

Supporting Information for

Inducing Fe 3*d* Electron Delocalization and Spin State Transition of FeN_4 Species Boosts Oxygen Reduction Reaction for Wearable Zinc Air Battery

Shengmei Chen[1, #], Xiongyi Liang[1, #], Sixia Hu[2, #], Xinliang Li[1], Guobin Zhang[1, *], Shuyun Wang[1], Longtao Ma[3], Chi-Man Lawrence Wu[1], Chunyi Zhi[1] Juan Antonio Zapien[1, *]

[1] Department of Materials Science and Engineering, City University of Hong Kong, Hong Kong SAR, 999077, P. R. China

[2] Sustech Core Research Facilities, Southern University of Science and Technology, 1088 Xueyuan Blvd., Shenzhen, Guangdong 518055, P. R. China

[3] Frontiers Science Center for Flexible Electronics, Institute of Flexible Electronics, Northwestern Polytechnical University, Xi'an 710072 P. R. China

[#] Shengmei Chen, Xiongyi Liang, and Sixia Hu contribute equally to this work.

*Corresponding authors. E-mail:: guobin.zhang@cityu.edu.hk (Guobin Zhang);: apjazs@cityu.edu.hk (Juan Antonio Zapien)

Supplementary Figures and Tables

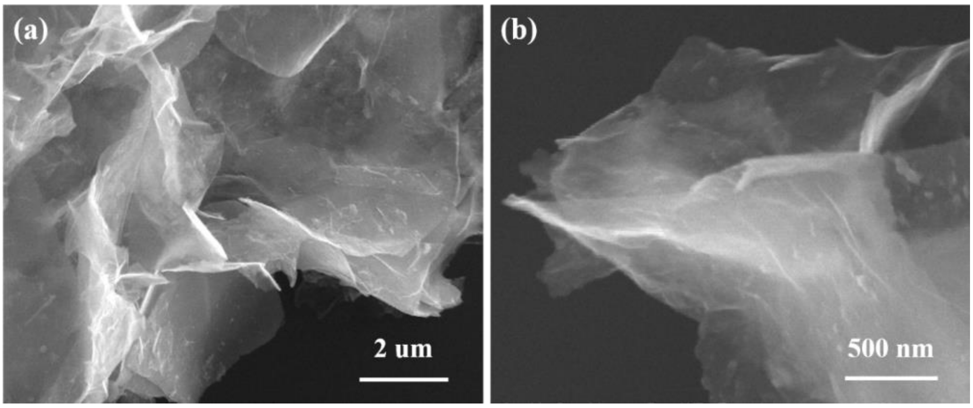

Fig. S1 SEM images of precursors of (**a**) FeN_4-Ti_3C_2, (**b**) and FeN_4-$Ti_3C_2S_x$ before carbonization

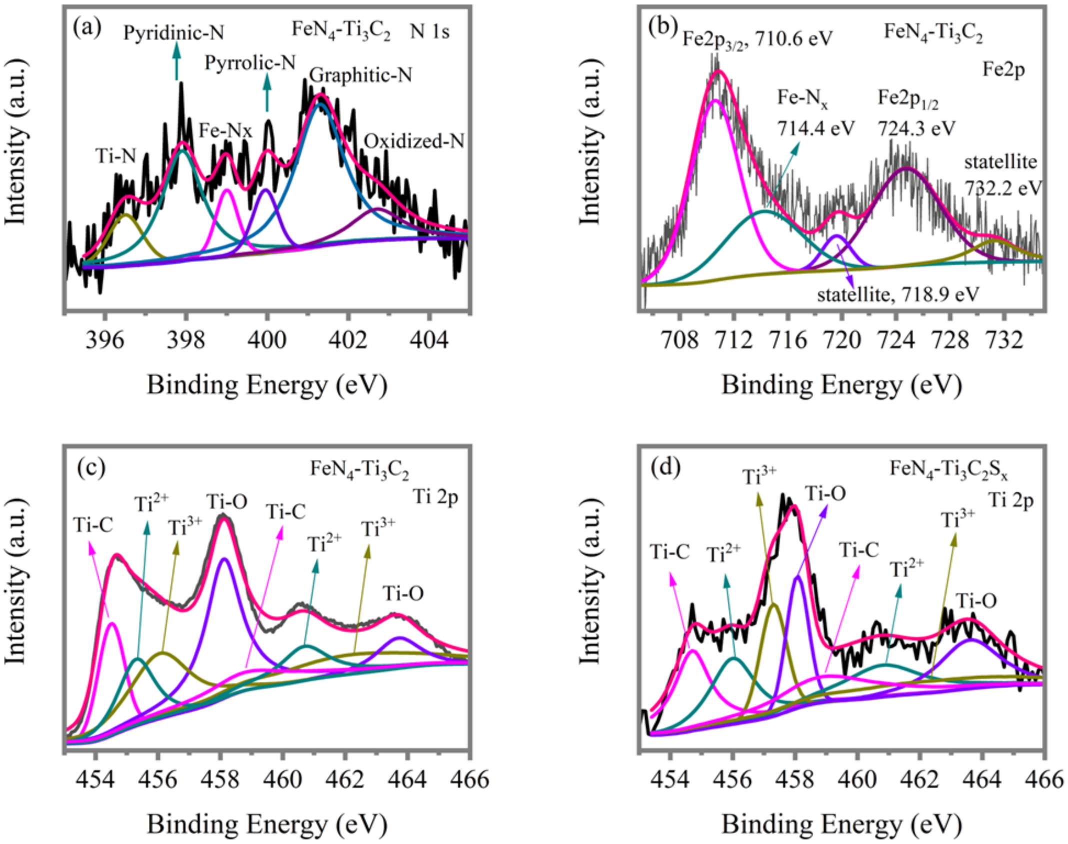

Fig. S2 High-resolution XPS of (**a**) N 1s, (**b**) Fe 2p, and (**c**) Ti 2p for sample FeN_4-Ti_3C_2. (**d**) High-resolution XPS of Ti 2p for sample FeN_4-$Ti_3C_2S_x$

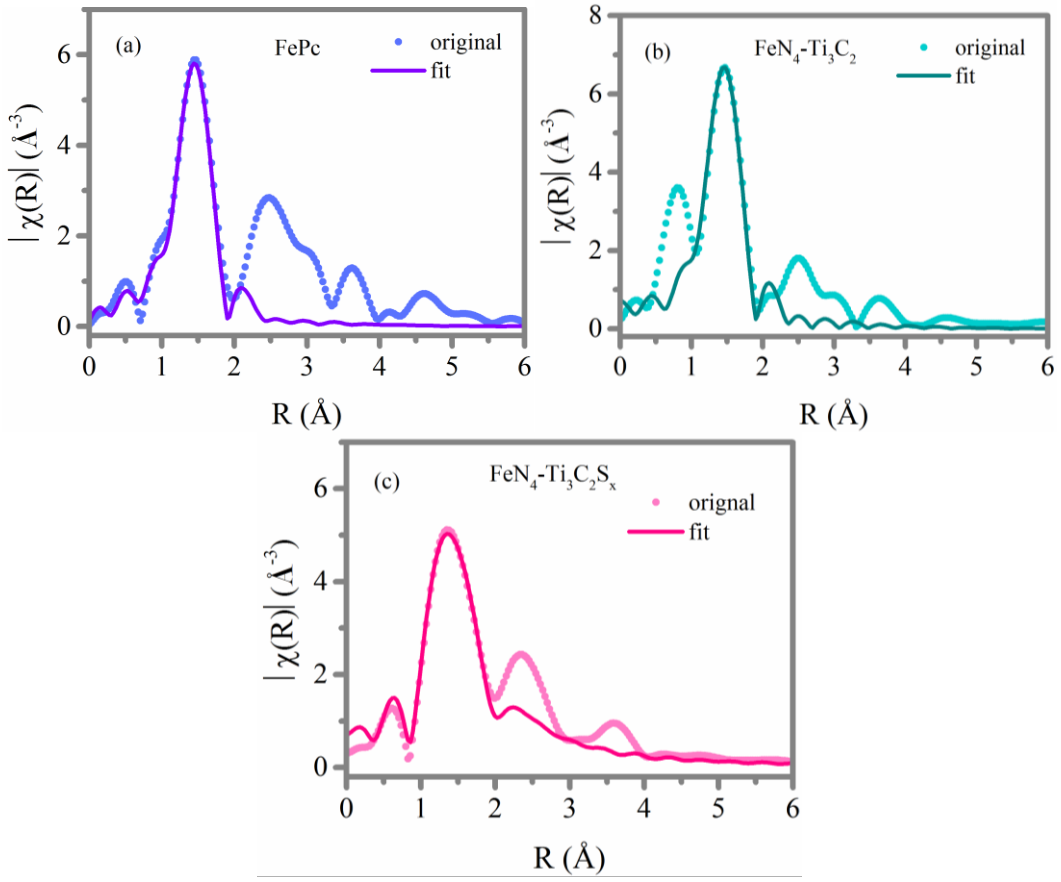

Fig. S3 Fourier Transforms (FT) together with the EXAFS fits of (**a**) commercial FePc, (**b**) FeN_4-Ti_3C_2, and (**c**) FeN_4-$Ti_3C_2S_x$

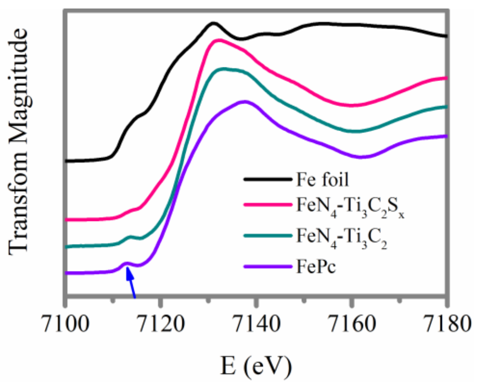

Fig. S4 Enlarged version of Figure 2f Fe K-edge XANES spectra of samples FeN_4-Ti_3C_2, and FeN_4-$Ti_3C_2S_x$, with Fe foil and FePc as references. The spectra have been offset in they-axis for clarity. The shoulder peak at ~ 7113.3 eV, arises from the 1 s → 4 P_z transition with simultaneous ligand to metal charge transfer, which has been assigned to the fingerprint of the square-planar FeN_4 moieties. Any distortion of the symmetry affects this transition intensely [S1]

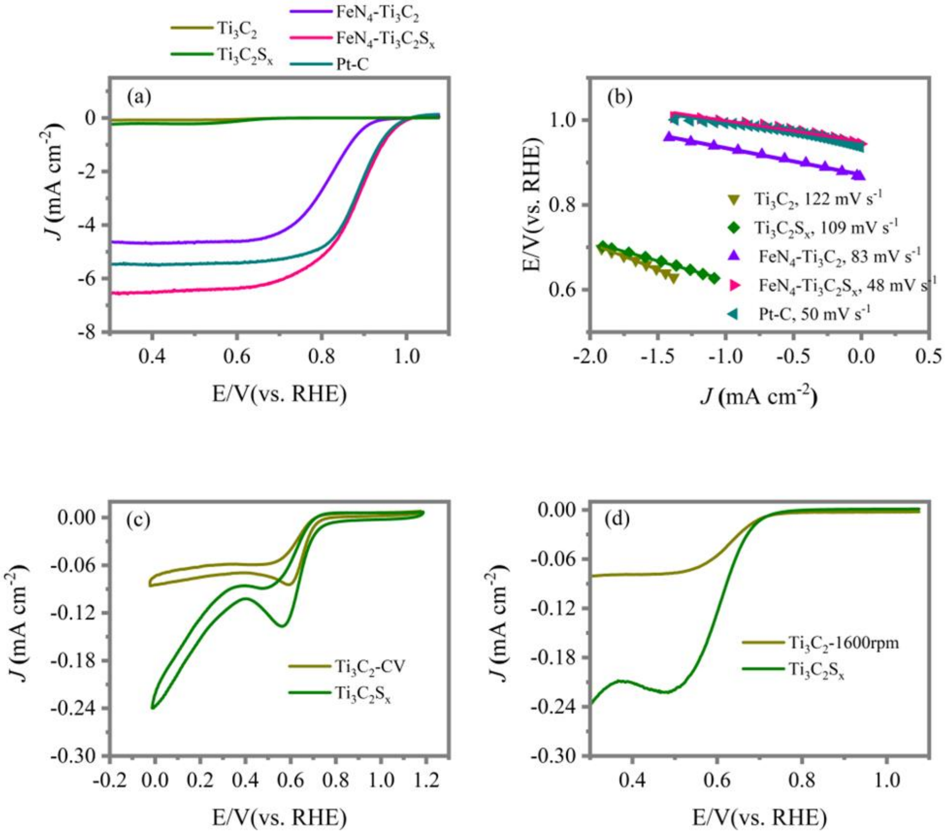

Fig. S5 (**a**) LSV curves of pristine Ti_3C_2, $Ti_3C_2S_x$, FeN_4 -Ti_3C_2, FeN_4 -$Ti_3C_2S_x$, and commercial Pt-C at 1600 rpm rotation speeds (**b**) The corresponding Tafel plots from LSV curves. We can see the Tafel slope of our samples follow the trend of Ti_3C_2 > $Ti_3C_2S_x$ > FeN_4-Ti_3C_2 >Pt-C >FeN_4-$Ti_3C_2S_x$, indicating the ORR kinetics follow the trend of Ti_3C_2 < $Ti_3C_2S_x$ < FeN_4-Ti_3C_2 < Pt-C < FeN_4-$Ti_3C_2S_x$. Enlarged version of CV (**c**) and LSV (**d**) curves of pristine Ti_3C_2 and $Ti_3C_2S_x$. We can see the onset, half wave potentials, and Tafel slope of $Ti_3C_2S_x$ (onset: 0.715 V, half-wave:0.640, Tafel slope, 109 mV s^{-1}) are similar to those of Ti_3C_2 (onset: 0.721 V, half-wave:0.645 V, Tafel slope, 122 mV s^{-1}), which suggests that the Ti_3C_2 with sulfur terminal nearly can not reduce oxygen itself in comparison toTi_3C_2 and FeN_4 -$Ti_3C_2S_x$. The enhanced catalytic activity of FeN_4 -$Ti_3C_2S_x$ is caused by the sulfur terminal MXene inducing the spin state transition of FeN_4 species and Fe 3*d* electron delocalization with d band center upshift

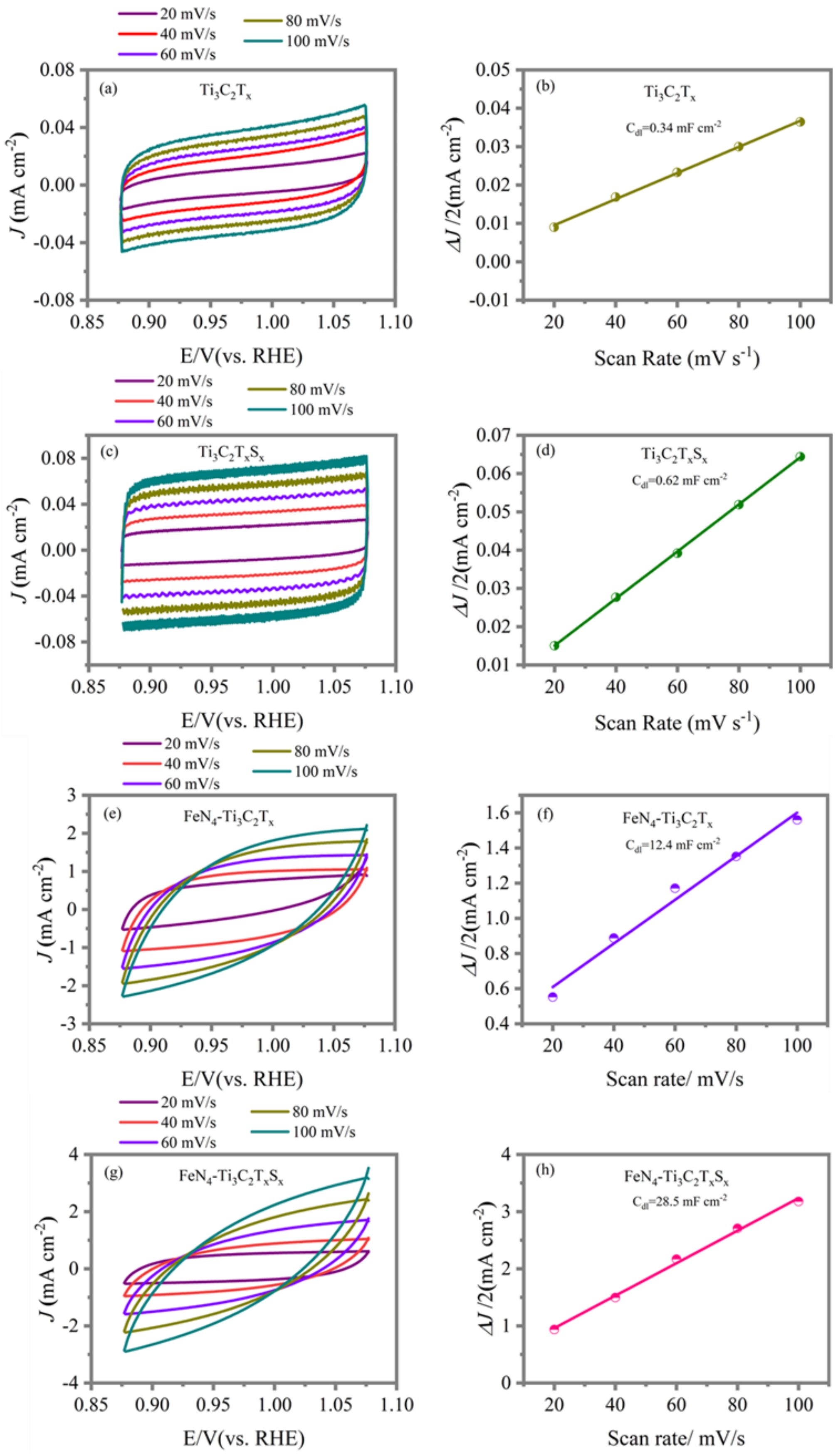

Fig. S6 CV curves in the region of -0.1-0.1 V at scan rate from 20 to 100 mV s^{-1} and corresponding liner fitting capacitive current vs. scan rates to estimate the C_{dl}: 0.34 mF cm^{-2} for Ti_3C_2 (**a, b**), 0.62 mF cm^{-2} for $Ti_3C_2S_x$ (**c, d**), 12.4 mF cm^{-2} for FeN_4-Ti_3C_2 (**e, f**), and 28.5 mF cm^2 for FeN_4-$Ti_3C_2S_x$ (**g, h**)

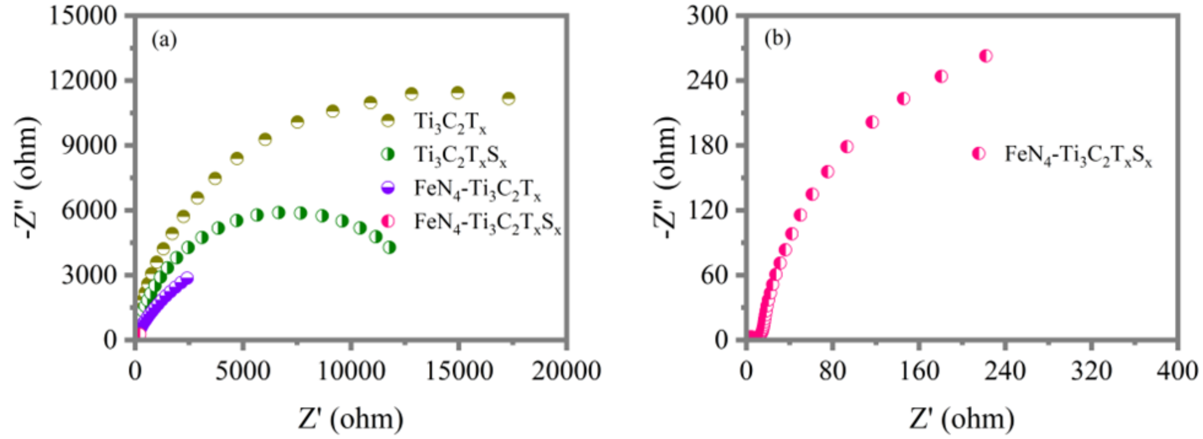

Fig. S7 (**a**) The A.C impedance plots for Ti_3C_2, $Ti_3C_2S_x$, FeN_4-Ti_3C_2, and FeN_4-$Ti_3C_2S_x$. (**b**) The enlarged version of A.C impedance plot for FeN_4-$Ti_3C_2S_x$

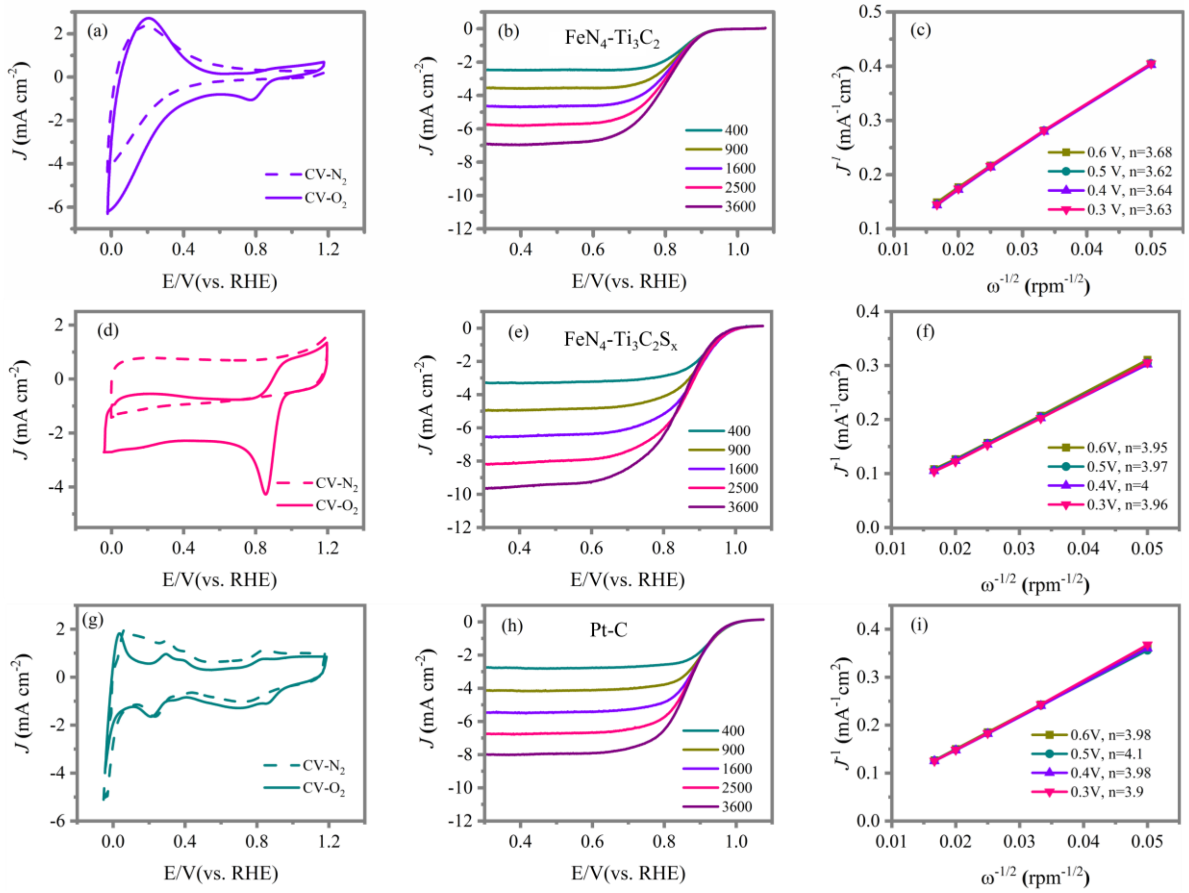

Fig. S8 Electrochemical characterization of (**a-c**) FeN_4-Ti_3C_2, (**d-f**) FeN_4-$Ti_3C_2S_x$, and (**g-i**) Pt-C catalysts. (**a, d, g**) CV curves of the electrocatalysts recorded at 100 mV s^{-1} in N_2 and O_2 saturated 0.1 M KOH solution; (**b, e, h**) corresponding LSV curves at the rotation speeds indicated; (**c, f, i**) corresponding K-L plots at the potentials indicated

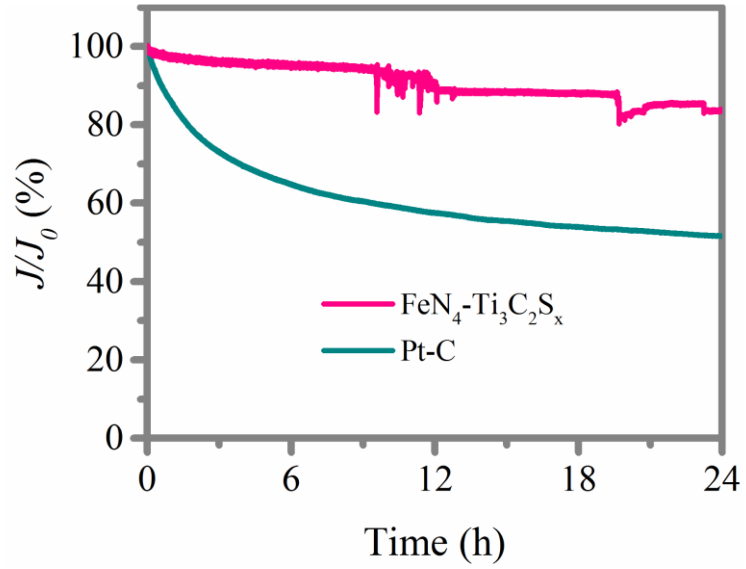

Fig. S9 Chronoamperometric stability tests of the FeN_4-$Ti_3C_2S_x$ and Pt-C catalyst at 1600 rpm at 0.7 V (vs. RHE) in O_2 saturated 0.1 M KOH solution. The stability performance loss of FeN_4 -$Ti_3C_2S_x$ after 24 hours study is mainly caused by the dissolution of Fe in the catalyst. We have conduct inductively coupled plasma optical emission spectrometry (ICP-OES) analysis to prove this and find the pristine purified electrolyte does not contain Fe element while the electrolyte after 24 hours cycling contain 6.24 ppm of Fe element

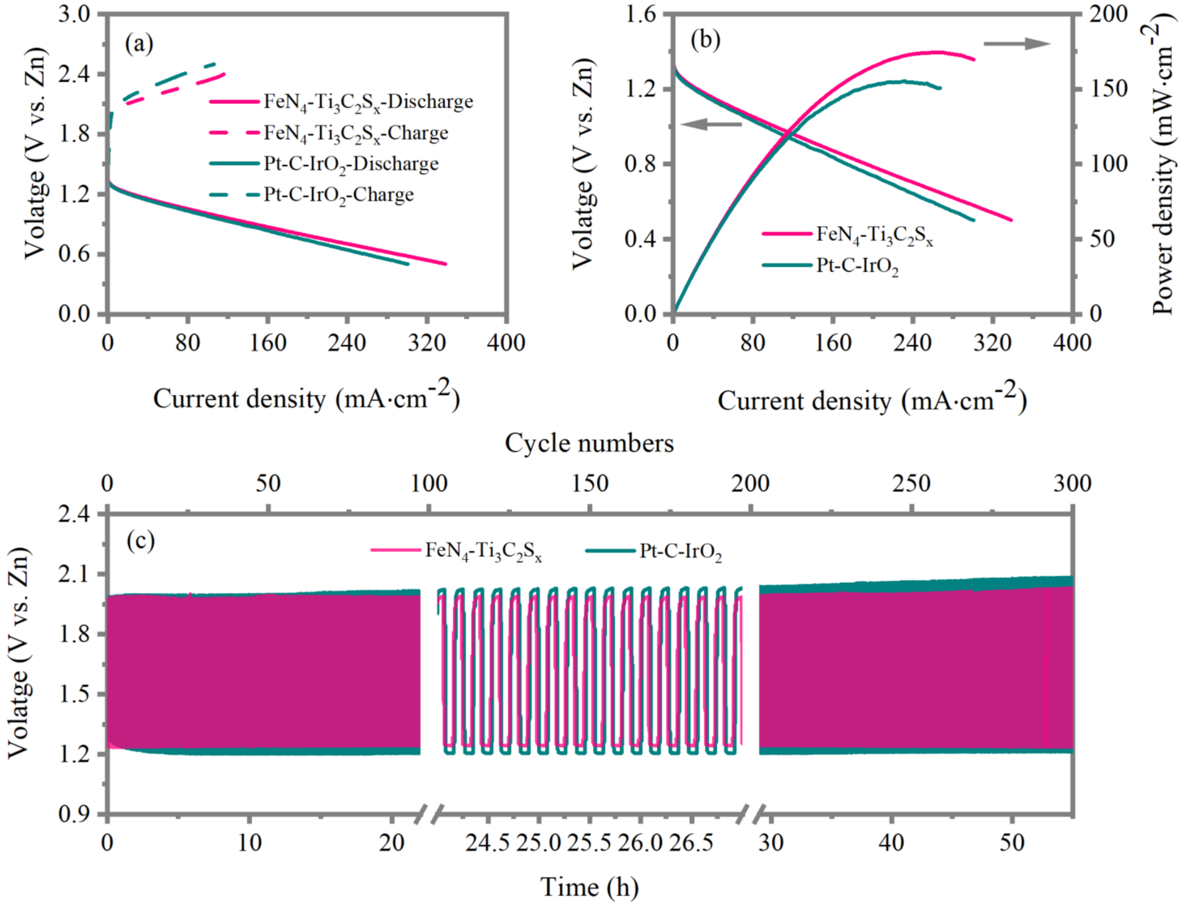

Fig. S10 Electrochemical performance of our developed FeN_4-$Ti_3C_2S_x$ and commercial Pt-C materials used as cathode electrocatalysts in aqueous ZAB. (**a**) Discharge-charge polarization curves; (**b**) discharge polarization and corresponding power density curves versus Zn electrode; (**c**) cycling tests at current density of 10 $mA{\cdot}cm^{-2}$

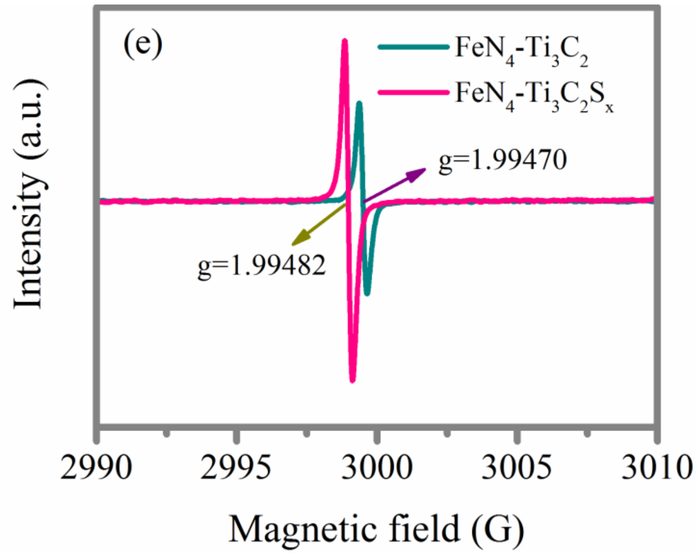

Fig. S11 X-band ESR spectra of samples FeN_4-Ti_3C_2 and FeN_4-$Ti_3C_2S_x$

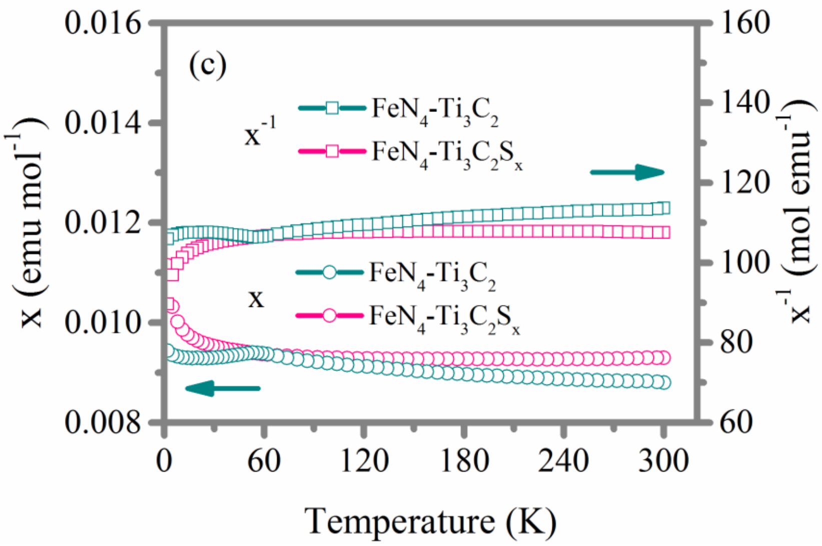

Fig. S12 Magnetic susceptibility x^{-1} and inverse magnetic susceptibility curves x of samples FeN_4 -Ti_3C_2, and FeN_4 -$Ti_3C_2S_x$

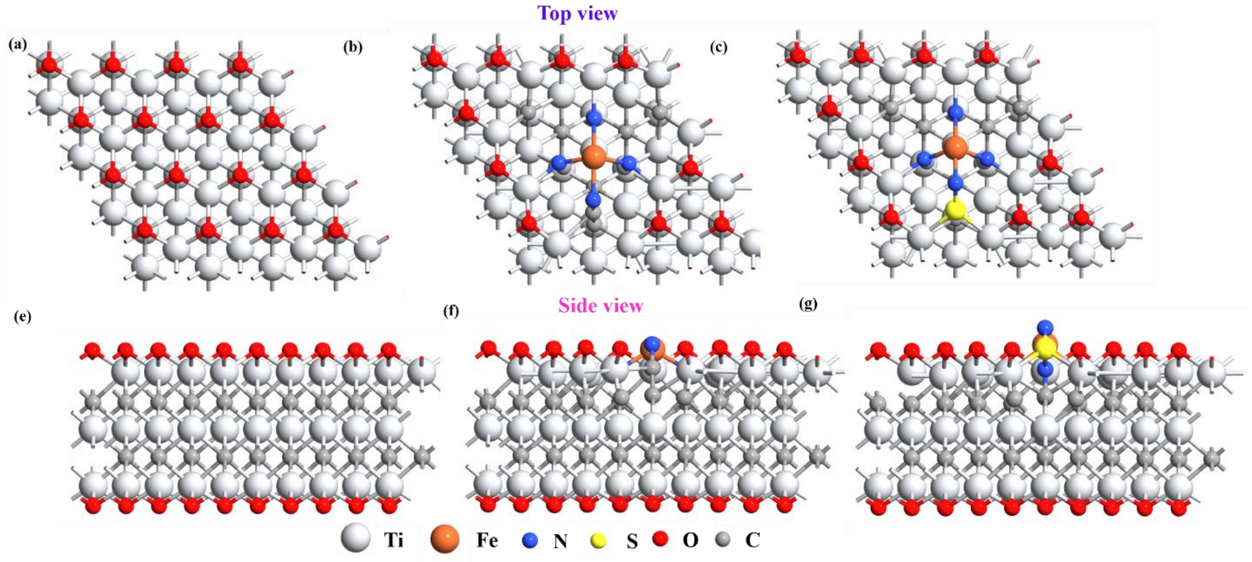

Fig. S13 Top view and side view of computational optimized atomic structures of pristine Ti_3C_2 (**a, e**), FeN_4-Ti_3C_2 (**b, f**), and FeN_4-$Ti_3C_2S_x$ (**c, g**)

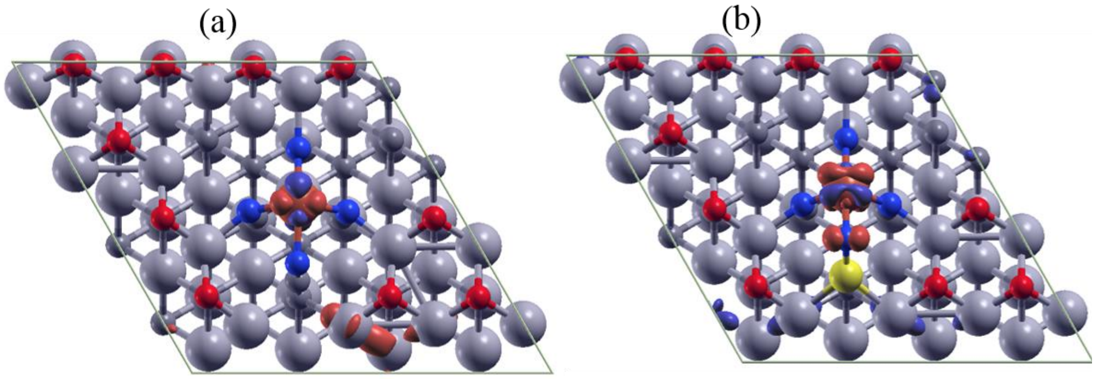

Fig. S14 Calculated spin density for (**a**) FeN_4-Ti_3C_2 and (**b**) FeN_4-$Ti_3C_2S_x$. The red/blue color isosurfaces represents spin up/down, respectively

Table S1 Ratio analysis of the peaks in XPS survey spectra of sample FeN_4-Ti_3C_2 and FeN_4-$Ti_3C_2S_x$

	% C	% Ti	% O	% S	% N	Fe %
FeN_4-Ti_3C_2	29.16	27.90	29.34	0	10.40	3.29
FeN_4-$Ti_3C_2S_x$	39.27	16.56	26.71	6.52	5.31	5.63

Table S2 Results of fitting EXAFS data for commercial FePc, FeN_4-Ti_3C_2, and FeN_4-$Ti_3C_2S_x$. Coordination number (N) and phase-corrected bond length (R) are shown for each interaction. Also shown are the Debye-Waller factor (σ2) and edge shifts (E_0)

Sample	Shell	N [a]	R (Å) [b]	σ^2 ($Å^2 \cdot 10^{-3}$) [c]	ΔE_0 (eV) [d]	*R* factor (%)
FePc	Fe-N	4	1.92	8.4	3.4	6.5
FeN_4-Ti_3C_2	Fe-N	4.1	1.97	1.2	5.3	5.9
FeN_4-$Ti_3C_2S_x$	Fe-N	4.1	1.98	5.3	8.2	5.7

[a] *N*: coordination numbers; [b] *R*: bond distance; [c] σ^2: Debye-Waller factors; [d] ΔE_0: the inner potential correction. *R* factor: goodness of fit. S_0^2 was set as 0.8 for Fe-N, which was derived from experimental EXAFS fitting of reference FePc via fixing CN as the known crystallographic value and applied to all samples.

Table S3 Comparison of ORR performance between FeN_4-$Ti_3C_2S_x$ electrocatalyst and recently reported electrocatalysts in the literatures

Electrocatalysts	E_{onset}/V vs. RHE	$E_{1/2}$/V vs. RHE	Loading Mass (ug cm^{-2})	Refs.
FeN_4-$Ti_3C_2S_x$	1.01	0.89	280	This work
FeN_4-Ti_3C_2	0.93	0.81	280	This work
Fe/OES	1.00	0.85	400	Angew. Chem. Int. Ed. **2020**, 132, 7454 –7459
Co-Co_3O_4@NAC	0.94	0.80	300	Appl. Catal. B Environ. **2020**, 260 1181882.
Mo SACs/N-C	-	0.83	810	Nano Energy **2020**, 67, 104288.
Co_3HITP_2	0.91	0.80	-	Angew. Chem. Int. Ed. **2020**, 59, 286 –294
Co-Nx/EPCF	0.95	0.82	-	J. Power Sources **2022**, 544, 231865

FeCo-N-HCN	0.98	0.86	-	Adv. Funct. Mater. **2021**, 2011289
NCAG/Fe−Cu	1.07	0.94	255	Angew. Chem. Int. Ed. **2022**, 61, e2022010
Fe,Mn/N-C	0.979	0.928	100	Nat. Commun. **2021**, 12, 1734
Fe-Co_2P@Fe-N-C	0.92	0.88	-	Small **2021**, 17, 2101
Cu/Zn-NC	0.98	0.83	250	Angew. Chem. Int. Ed. **2021**, 60, 14005
O–Co–N/C	-	0.85	120	Adv. Funct. Mater. **2022**, 32, 2200

Supplementary Reference

[S1] Q. Jia, N. Ramaswamy, H. Hafiz, U. Tylus, K. Strickland et al., Experimental observation of redox-induced Fe–N switching behavior as a determinant role for oxygen reduction activity. ACS Nano **9**(12), 12496-12505 (2015). https://doi.org/10.1021/acsnano.5b05984
